# The Unfolded-Protein Response Triggers the Arthropod Immune Deficiency Pathway

**DOI:** 10.1128/mbio.00703-22

**Published:** 2022-07-18

**Authors:** Lindsay C. Sidak-Loftis, Kristin L. Rosche, Natasha Pence, Jessica K. Ujczo, Joanna Hurtado, Elis A. Fisk, Alan G. Goodman, Susan M. Noh, John W. Peters, Dana K. Shaw

**Affiliations:** a Program in Vector-borne Disease, Department of Veterinary Microbiology and Pathology, Washington State University, Pullman, Washington, USA; b Institute of Biological Chemistry, Washington State University, Pullman, Washington, USA; c United States Department of Agriculture, Agricultural Research Service, Animal Disease Research Unit, Pullman, Washington, USA; d School of Molecular Biosciences, Washington State University, Pullman, Washington, USA; University of Texas Medical Branch at Galveston; McGovern Medical School

**Keywords:** *Ixodes scapularis*, immune deficiency pathway, unfolded-protein response, *Borrelia burgdorferi*, *Anaplasma phagocytophilum*, tick-borne disease, vector immunity

## Abstract

The insect immune deficiency (IMD) pathway is a defense mechanism that senses and responds to Gram-negative bacteria. Ticks lack genes encoding upstream components that initiate the IMD pathway. Despite this deficiency, core signaling molecules are present and functionally restrict tick-borne pathogens. The molecular events preceding activation remain undefined. Here, we show that the unfolded-protein response (UPR) initiates the IMD network. The endoplasmic reticulum (ER) stress receptor IRE1α is phosphorylated in response to tick-borne bacteria but does not splice the mRNA encoding XBP1. Instead, through protein modeling and reciprocal pulldowns, we show that *Ixodes* IRE1α complexes with TRAF2. Disrupting IRE1α-TRAF2 signaling blocks IMD pathway activation and diminishes the production of reactive oxygen species. Through *in vitro*, *in vivo*, and *ex vivo* techniques, we demonstrate that the UPR-IMD pathway circuitry limits the Lyme disease-causing spirochete Borrelia burgdorferi and the rickettsial agents Anaplasma phagocytophilum and A. marginale (anaplasmosis). Altogether, our study uncovers a novel linkage between the UPR and the IMD pathway in arthropods.

## INTRODUCTION

Arthropod-borne diseases continue to be a substantial source of morbidity and mortality worldwide (1). Factors influencing the ability of arthropods to harbor and transmit pathogens are incompletely understood, although progress on this front has been made in recent years. Arthropod immunity is an important force in shaping vector competency (2–9). For example, humoral defense networks such as the immune deficiency (IMD) pathway recognize and restrict invading microbes. As classically defined in Drosophila melanogaster, IMD pathway signaling events are similar to the tumor necrosis factor receptor (TNFR) pathway in mammals but instead respond to the Gram-negative bacterial pathogen-associated molecular pattern (PAMP) diaminopimelic acid (DAP)-type peptidoglycan (PGN) (10, 11). Pathway-initiating receptors PGRP-LC and PGRP-LE (peptidoglycan recognition proteins LC and LE) recruit adapter molecules IMD and FADD (Fas-associated protein with death domain) (12, 13), the latter pairing with DREDD (death-related ced-3/Nedd2-like protein) (14), which cleaves IMD. The E3 ubiquitin ligase IAP2 (inhibitor of apoptosis 2) and E2-conjugating enzymes Bendless, Uev1a, and Effette then promote K63 polyubiquitylation of IMD (10, 11, 15). The resulting signaling scaffold leads to cleavage of the NF-κB signaling molecule Relish, which translocates to the nucleus and promotes antimicrobial peptide (AMP) expression (11, 15).

Significant advances in characterizing arthropod immunity have been possible owing to the insect model organism *Drosophila*. However, deviations from classically defined fly immunity have been reported. For example, some IMD pathway components are not found in the genomes of arachnids (e.g., mites, spiders, etc.) or several hemimetabolous insects, such as lice, bedbugs, psyllids, squash bugs, and whiteflies (16–28). Triatomine bugs recently had many IMD pathway components identified but are missing the gene encoding IMD itself (29–31). Ixodes scapularis ticks lack genes encoding upstream regulators of the IMD pathway, including transmembrane *PGRPs*, *imd*, and *fadd* (16, 28, 32, 33). Despite the absence of upstream regulators, core IMD signaling molecules are active against infection (30–34). Activity of the *Ixodes* IMD pathway hinges on Bendless, Uev1a, XIAP (X-linked inhibitor of apoptosis), p47, Relish, and the negative regulator Caspar, which functionally restricts the tick-borne pathogens Borrelia burgdorferi (Lyme disease) and Anaplasma phagocytophilum (granulocytic anaplasmosis) (5, 32, 33, 35). In the absence of classically defined pathway initiators, functionality of the core IMD cascade suggests that an alternative mode of activation exists.

Cellular stress responses are well conserved across eukaryotes and respond to adverse environmental conditions, such as infection (36–45). Here, we demonstrate that a stress-response network, the unfolded-protein response (UPR), initiates the IMD pathway in I. scapularis ticks. B. burgdorferi and A. phagocytophilum activate the endoplasmic reticulum (ER) stress receptor IRE1α (inositol-requiring enzyme 1α), which pairs with a TRAF2-like (TNF receptor associated factor 2-like) signaling molecule (here referred to as *Ixodes* TRAF2). Through molecular modeling, biochemical interactions, pharmacological manipulations, and RNA interference (RNAi), we show that the *Ixodes* IRE1α-TRAF2 axis functionally restricts B. burgdorferi and A. phagocytophilum in ticks, induces the IMD pathway NF-κB factor Relish, and initiates production of antimicrobial effectors. IRE1α-TRAF2 signaling also restricts the cattle pathogen Anaplasma marginale in Dermacentor andersoni ticks. Collectively, we show a fundamentally distinct mode of IMD pathway activation that explains how core signaling is activated independent of canonical upstream regulators.

## RESULTS

### The *Ixodes* UPR responds to tick-borne pathogens and restricts bacterial colonization.

**FIG 1:**
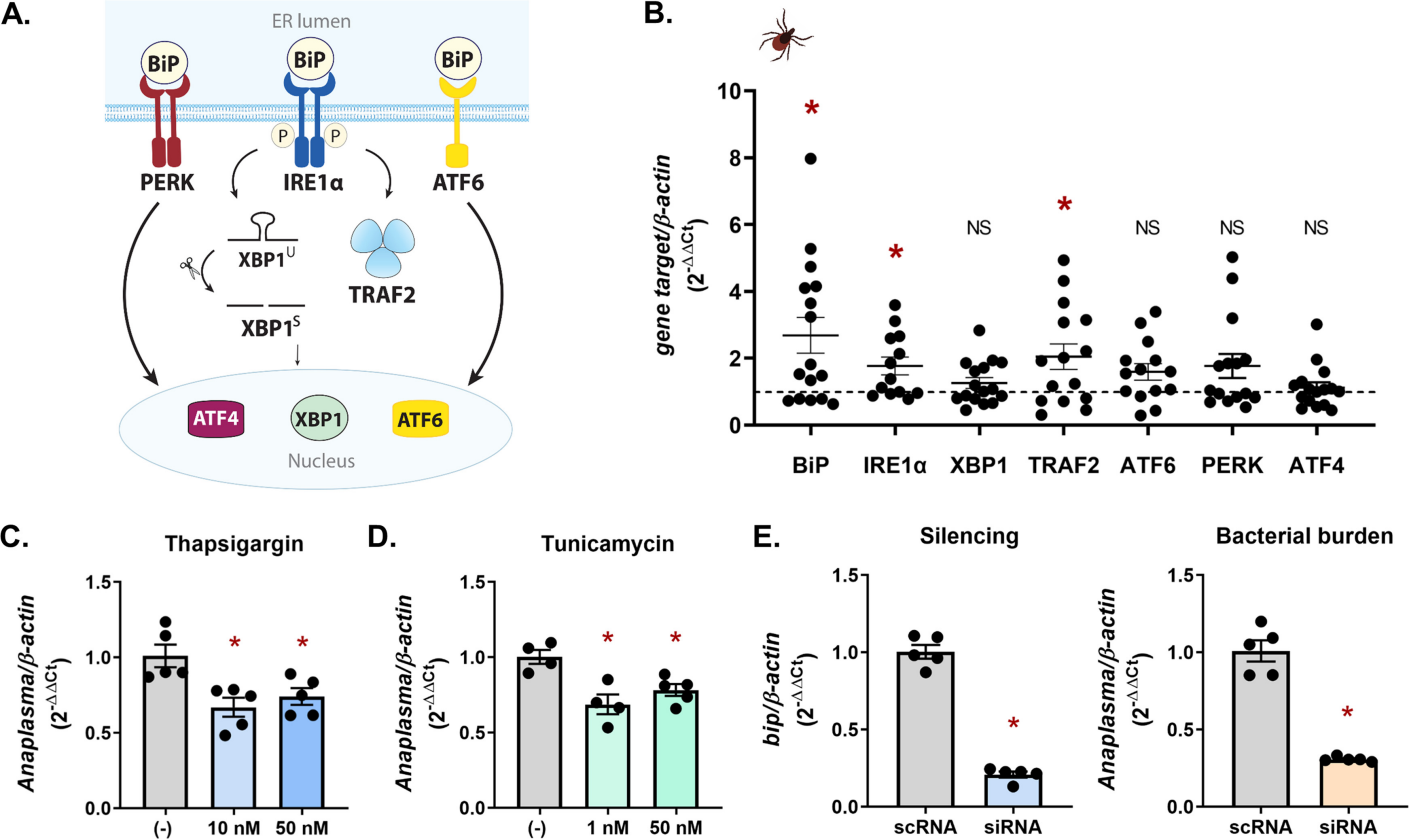
The tick UPR responds to and restricts bacterial colonization. (A) Graphic representation of the UPR in mammals. (B) UPR gene expression in A. phagocytophilum*-*infected I. scapularis nymphs relative to uninfected controls (dotted line). Each point is representative of 1 nymph. Gene expression was quantified by qRT-PCR. (C to E) ISE6 cells (1 × 10^6^) were infected with A. phagocytophilum at an MOI of 50 for 18 h following a 24-h treatment with either (C) thapsigargin, (D) tunicamycin, or (E) siRNA targeting the negative regulator *bip*. Gene silencing and A. phagocytophilum load (16S rRNA gene) were measured by qRT-PCR. Data are representative of 5 biological replicates with least two technical replicates; error bars show SEM. *, *P* < 0.05 (Student's *t* test). scRNA, scrambled RNA; siRNA, small interfering RNA; NS, not significant. See also Fig. S1.

The absence of IMD pathway-initiating molecules led us to hypothesize that the core signaling components may be induced through cross talk with other molecular circuits. A response network that is capable of detecting pathogen colonization is the UPR (36–38, 43, 44, 46–49). The UPR is a highly conserved cellular stress response that is activated when the ER is under stress, such as during infection (36–38). Infection exerts stress on the host system (50), and for this reason, cellular stress responses are tightly intertwined with innate immunity (42, 43, 51–54). The UPR is activated through the transmembrane receptors IRE1α, PERK (PKR-like ER kinase), and ATF6 (activating transcription factor 6). In a nonstressed state, the sensor molecule BiP (binding immunoglobulin protein) keeps all receptors inactive by binding to them (36–38) (Fig. 1A). ER stress causes BiP to disassociate from UPR receptors, allowing downstream signaling to ensue (36, 55–57). This also results in upregulated expression of many UPR components, including BiP, with the goal of restoring cellular homeostasis (36–38, 42, 58–59, 140). To evaluate whether tick-borne pathogens induce the UPR in I. scapularis, we quantified UPR-associated gene expression in A. phagocytophilum-infected nymphs. Specifically, we evaluated expression levels of BiP, the three UPR receptors (IRE1α, PERK, and ATF6), and molecules associated with the IRE1α pathway, XBP1 and TRAF2. Relative to uninfected ticks (Fig. 1B, dotted baseline), significant increases were observed with *BiP*, *ire1α*, and *traf2*, suggesting that the tick UPR responds to infection (Fig. 1B).

**FIG 2:**
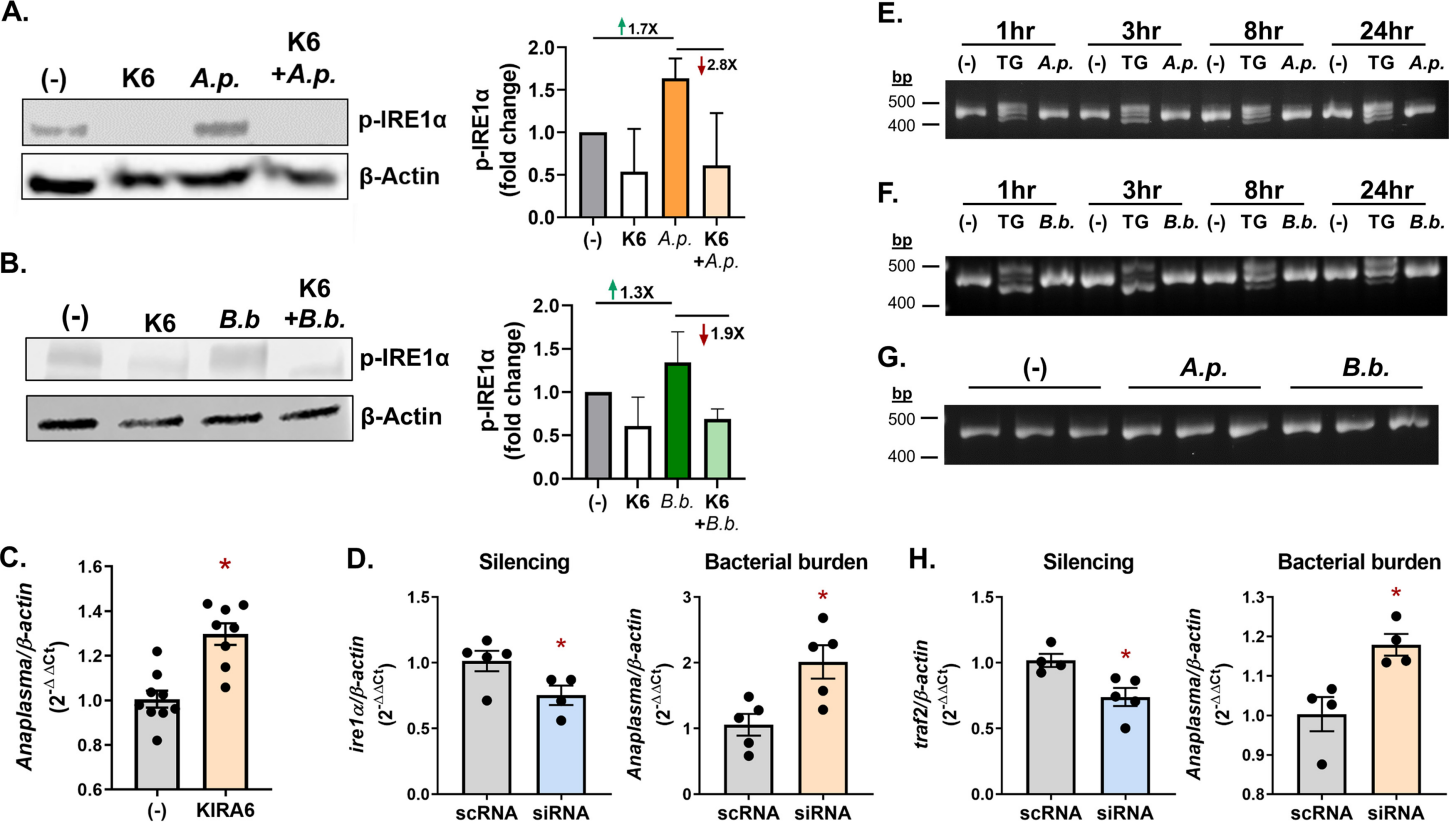
The IRE1α branch of the UPR is induced by tick-borne pathogens through TRAF2. (A and B) Phosphorylated IRE1α immunoblot against ISE6 (1 × 10^6^) cells treated with the IRE1α inhibitor KIRA6 (K6; 1 h), infected with A. phagocytophilum (*A.p.*) or B. burgdorferi (*B.b.*) for 24 h, or treated in combination (1 h KIRA6 pretreatment followed by A. phagocytophilum or B. burgdorferi infection for 24 h). Immunoblots are representative of 2 biological replicates. Protein expression differences were quantified by ImageJ and are expressed as a ratio of phosphorylated IRE1α (~110 kDa) to the internal loading control, β-actin (45 kDa). (C, D, and H) ISE6 cells were treated with (C) the IRE1α inhibitor KIRA6 (1 h) or (D and H) siRNAs to silence gene expression prior to A. phagocytophilum (MOI, 50) infection for 18 h. Gene silencing and A. phagocytophilum burden were measured by qRT-PCR. The data are representative of at least two technical replicates; error bars show SEM. *, *P* < 0.05 (Student's *t* test). (E and F) ISE6 cells (1 × 10^6^) were either untreated (−), stimulated with 0.5 μM thapsigargin (TG), infected with A. phagocytophilum (MOI, 50) or infected with B. burgdorferi (MOI, 50) for the indicated times. (G) Replete I. scapularis nymphs were fed either on uninfected mice (−), A. phagocytophilum-infected or B. burgdorferi-infected mice. (E to G) cDNA was synthesized from RNA and used to evaluate *xbp1* splicing by PCR. Samples were analyzed on a 3% agarose gel. scRNA, scrambled RNA; siRNA, small interfering RNA. See also Fig. S1.

10.1128/mbio.00703-22.1 FIG S1 (Related to Fig. 2.) UPR molecules are conserved in the I. scapularis genome. (A to D) Amino acid sequence alignment of BiP, IRE1α, TRAF2, and XBP1 between H. sapiens and I. scapularis. Alignments were created using available sequences from NCBI imported into Jalview. Shaded regions indicate amino acid physiochemical property conservation. Good conservation between sequences was observed for (A) BiP, (B) the IRE1α protein kinase domain (light blue box) and RNase domain (grey box), (C) the TRAF-type zinc finger domain of TRAF2 (yellow box), and (D) the basic region leucine zipper (bZIP) domain in XBP1 (blue box). (E) Nucleotide sequence for I. scapularis *xbp1* mRNA. The internal intron that is spliced by the RNase domain of IRE1α is underlined in blue. Cleavage sites are indicated by red lettering, and black arrows indicate primer sites used to confirm *xbp1* splicing by PCR. Download FIG S1, TIF file, 2.7 MB. Copyright © 2022 Sidak-Loftis et al. 2022 Sidak-Loftis et al. https://creativecommons.org/licenses/by/4.0/ This content is distributed under the terms of the Creative Commons Attribution 4.0 International license.

To determine how the UPR impacts pathogen survival in ticks, we used pharmacological inducers or RNAi with the ISE6 I. scapularis cell line. Tick cells were treated with low doses of either thapsigargin or tunicamycin to induce ER stress prior to A. phagocytophilum infection. Thapsigargin inhibits the sarco/endoplasmic reticulum Ca^2+^ ATPase (SERCA), which decreases calcium levels in the ER (60). Tunicamycin blocks N-linked glycosylation, leading to an increase of misfolded proteins (61). Both treatments resulted in significantly less A. phagocytophilum (Fig. 1C and D). We also used an RNAi-based approach to overactivate the UPR by decreasing expression of the negative regulator BiP. In agreement with pharmacological induction, transcriptional silencing of BiP caused a decrease in A. phagocytophilum colonization (Fig. 1E). Altogether, this demonstrates that A. phagocytophilum induces the UPR in ticks, which functionally restricts bacterial colonization and survival.

### Infection induces IRE1α activation, but not XBP1.

Transcripts induced by A. phagocytophilum are associated with the IRE1α signaling axis (Fig. 1A and B), which is the most conserved branch of the UPR among eukaryotes (62). When activated, IRE1α autophosphorylates and either splices the mRNA *xbp1* (X-box binding protein 1) or signals through TRAF2 (36, 37, 46, 57) (Fig. 1A). Unspliced *xbp1* mRNA (*xbp1^U^*) is held in an inactive state in the cytoplasm by forming a hairpin structure that inhibits translation. The RNase domain of IRE1α splices an internal intron from *xpb1^U^*, allowing it to be translated into a protein that functions as a transcription factor (57, 63–66) (Fig. 1A). Alternatively, IRE1α can recruit the signaling molecule TRAF2 to produce proinflammatory responses through NF-κB signaling (36–38, 46). We aligned mammalian sequences from the IRE1α pathway with tick homologs and observed sequence similarity with BiP, IRE1α, XBP1, and TRAF2 (Fig. S1A to D). Notably, the IRE1α kinase domain, RNase domain, and the activity-inducing phosphoserine (Fig. S1B) were well conserved with human sequences. Given this sequence conservation, we used an antibody against human phosphorylated IRE1α to examine the posttranslational activation status of IRE1α in ticks. Upon treatment with the UPR inducers thapsigargin and tunicamycin, increased IRE1α phosphorylation was observed in ISE6 tick cells by immunoblotting, as expected (Fig. S2A). A. phagocytophilum and B. burgdorferi also induced IRE1α phosphorylation in ISE6 cells, indicating that infection induces receptor activation (Fig. 2A and B). A small-molecule inhibitor, KIRA6 (67), successfully blocked IRE1α phosphorylation during infection (Fig. 2A and B). Inhibiting IRE1α phosphorylation caused significantly increased infection in tick cells (Fig. 2C). Similarly, knocking down the expression of *ire1α* through RNAi also increased A. phagocytophilum bacterial burden (Fig. 2D; Fig. S2B). These data show that IRE1α signaling in ticks is activated by infection and restricts bacterial colonization *in vitro*.

10.1128/mbio.00703-22.2 FIG S2 (Related to Fig. 2.) IRE1α phosphorylation is affected during ER stress and RNAi manipulation in tick cells. (A and B) Phosphorylated IRE1α immunoblot against ISE6 (1 × 10^6^) cells treated with (A) the ER stress inducers tunicamycin (Tu; 50 nM) and thapsigargin (TG; 50 nM) or (B) siRNAs to silencing gene expression of *ire1α* for 24 h prior to infection with A. phagocytophilum. The immunoblot is representative of 2 biological replicates. Protein expression differences were quantified by ImageJ and are expressed as a ratio of phosphorylated IRE1α (~110 kDa) to the internal loading control, β-actin (45 kDa). Download FIG S2, TIF file, 0.9 MB. Copyright © 2022 Sidak-Loftis et al. 2022 Sidak-Loftis et al. https://creativecommons.org/licenses/by/4.0/ This content is distributed under the terms of the Creative Commons Attribution 4.0 International license.

To delineate the signaling events downstream from IRE1α, *xbp1^U^* was next examined in infected ISE6 cells. Primers flanking the *xbp1* intron (Fig. S1E) were used to differentiate spliced and unspliced transcripts by PCR. Unspliced *xbp1^U^* migrates as a single 459-bp band. In contrast, spliced *xbp1^S^* presents as a trimer on an agarose gel, consisting of spliced transcripts (*xbp1^S^*, 434 bp), unspliced transcripts (*xbp1^U^*), and an *xbp1^U^*-*xbp1^S^* heterodimer that is an artifact of PCR and migrates slightly higher. Spliced *xbp1^S^* was observed in thapsigargin-treated tick cells under all conditions. In contrast, neither A. phagocytophilum or B. burgdorferi induced *xbp1^U^* splicing at any time points *in vitro* (Fig. 2E and F). We next probed *in vivo* samples from replete I. scapularis nymphs that were fed on either uninfected mice or mice infected with A. phagocytophilum or B. burgdorferi. Across all samples, *xbp1^U^* remained unspliced (Fig. 2G). These results indicate that although the tick IRE1α is activated by infection and restricts bacterial burden, this phenotype is not carried out through XBP1 activity.

Since XBP1 is not responsive to infection, we sought to determine if TRAF2 has a functional role during pathogen colonization. Reducing the expression of *traf2* through RNAi in *Ixodes* ISE6 cells caused a significant increase in A. phagocytophilum (Fig. 2H), correlating with the phenotype observed when *ire1α* transcripts were silenced (Fig. 2D). These data, together with upregulated *traf2* expression in A. phagocytophilum-infected I. scapularis nymphs (Fig. 1B), led us to further interrogate whether IRE1α signals through TRAF2 to restrict pathogen colonization.

### IRE1α interfaces with TRAF2 in I. scapularis ticks.

Aligning sequences from humans and ticks reveals that the *Ixodes* TRAF2 is fundamentally unique compared to the mammalian homolog (Fig. S3A). The *Ixodes* TRAF2 lacks a RING (really interesting new gene) domain that is necessary for ubiquitin ligase activity (68). The *Ixodes* TRAF2 also has a reduced TRAF-N domain, which is responsible for bridging interactions with other proteins (68). Given these differences, we performed homology modeling and a prediction-driven docking approach (69) with the I. scapularis IRE1α and TRAF2 proteins to gain insight into how they interact. BLAST was used to identify the human TRAF2 crystal structure (68) (PDB code 1CA9) as a modeling template for *Ixodes* TRAF2. The modeled form of the *Ixodes* TRAF2 C-terminal region features part of a coiled-coil domain and the highly conserved TRAF-C domain (Fig. S3B). In addition, the homology model is a trimer where the coiled-coil domain is a single alpha helix and the TRAF-C domain forms an eight-stranded antiparallel β-sandwich. Next, the human IRE1α crystal structure (72) (PDB code 6URC) was identified by BLAST as a homology template for modeling the cytosolic RNase/kinase domain of I. scapularis IRE1α. The structure was modeled in the active-state quaternary structure proposed to be necessary for autophosphorylation and RNase activity (73) (Fig. S3C and D).

**FIG 3:**
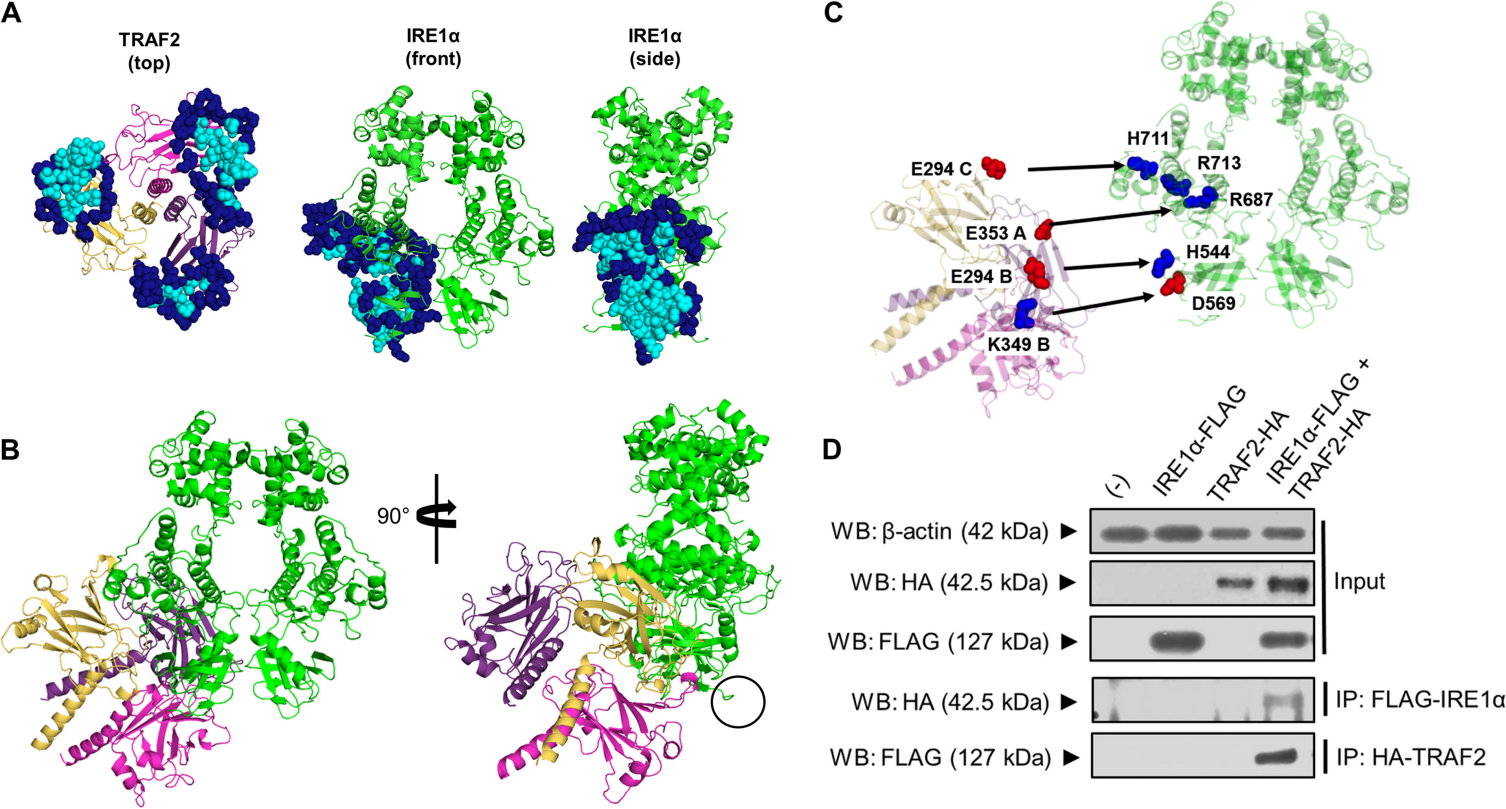
*Ixodes* IRE1α-TRAF2 molecular interactions. (A) Interfaces assigned by CPORT for the *Ixodes* TRAF2 trimer and IRE1α homology models. Active central (cyan) and passive peripheral (navy blue) residues, shown as spheres, were used to filter the docking solutions in HADDOCK 2.2. (B) The final model of docking between *Ixodes* TRAF2 and IRE1α places TRAF2 away from the dimer interface and the C terminus of IRE1α (black circle), which anchors IRE1α to the ER. (C) Salt bridges were determined between all three chains of *Ixodes* TRAF2 and IRE1α with a measured distance between 2.7 and 2.8 Å. Negatively charged Asp and Glu residues (red spheres) pair with positively charged Lys, Arg, and His residues (blue spheres). (D) Immunoprecipitation (IP) analysis followed by Western blotting (WB) showing interaction between FLAG-tagged *Ixodes* IRE1α and HA-tagged *Ixodes* TRAF2 expressed in HEK 293T cells. WB results are representative of two biological replicates. See also Fig. S3.

10.1128/mbio.00703-22.3 FIG S3 (Related to Fig. 3.) *Ixodes* IRE1α and TRAF2 homology models. (a) Domain comparison between human TRAF2 and *Ixodes* TRAF2 proteins. Orange, RING; green, zinc finger; blue, TRAF N domain; red, TRAF C domain. (B) The *Ixodes* TRAF2 homology model is a trimer with three chains labeled A (purple), B (magenta), and C (yellow). Part of the coiled-coil domain is modeled as a single alpha helix, and the TRAF-C domain forms an eight-stranded antiparallel β-sandwich. (C) *Ixodes* IRE1α homology model of the dimer RNase/kinase domain. The kinase region consists of an N lobe (yellow) and C lobe (aqua). (D) Residues of the kinase-extension nuclease (KEN) domain (top) and the kinase domain (bottom) are predicted to participate in salt bridge formation and dimerization. Residues at the KEN domain interface predicted to form salt bridges are shown in the top panel. Residues at the nucleotide binding pocket coordinate MgADP and are conserved with human IRE1α (middle). Salt bridge-forming residues at the kinase domain are predicted to participate in IRE1α dimerization (bottom). Download FIG S3, TIF file, 2.7 MB. Copyright © 2022 Sidak-Loftis et al. 2022 Sidak-Loftis et al. https://creativecommons.org/licenses/by/4.0/ This content is distributed under the terms of the Creative Commons Attribution 4.0 International license.

We then modeled the *Ixodes* IRE1α-TRAF2 complex using a prediction-driven docking approach (69). This tactic combines the utility of interface prediction with *ab initio* docking and is a useful alternative to *ab initio* docking alone when protein-protein complex formation is being examined. CPORT (consensus prediction of interface residues in transient complexes) (69) was used to assign active and passive residues at the interface of the trimeric TRAF-C domains and the RNase/kinase domain of IRE1α (Fig. 3A). Residues were then used to filter the docking process by HADDOCK 2.2 (74), which optimizes residue conformations at the interface before proceeding to refinement. The docking model places the trimeric TRAF2 interface at the kinase domain of IRE1α with a buried surface area of 3,262.16 Å^2^ (Fig. 3B). Importantly, trimeric TRAF2 is positioned in a manner that does not interfere with the IRE1α dimer interface and is away from the C-terminal transmembrane domain (Fig. 3B, circled) that anchors IRE1α to the ER (Fig. 3B). Five salt bridge interactions were identified that define how the TRAF2 trimer is positioned onto the kinase domain of IRE1α (Fig. 3C). Each chain of TRAF2 participates in salt bridge interactions with the kinase domain of IRE1α. Therefore, the oligomeric state of TRAF2 seems to play an important role in docking specificity with the RNase/kinase domain of IRE1α. Altogether, *in silico* docking analyses with *Ixodes* IRE1α and TRAF2 suggest that these two molecules can directly interface with one another.

To experimentally validate that IRE1α and TRAF2 specifically interact, we used a human embryonic kidney (HEK) 293T cell transfection system with plasmids expressing *Ixodes* IRE1α and TRAF2 fused to affinity tags (Fig. 3D). Recombinant protein expression was confirmed by immunoblotting transfected cells with antibodies for FLAG and hemagglutinin (HA) tags (IRE1α-FLAG and TRAF2-HA). When *Ixodes* IRE1α and TRAF2 are coexpressed, immunoprecipitation with antibodies against the FLAG tag demonstrates that IRE1α specifically pulls down TRAF2 and vice versa (Fig. 3D). Altogether, these data demonstrate that *Ixodes* IRE1α and TRAF2 directly and specifically interact.

### *Ixodes* IRE1α and TRAF2 restrict *in vivo* bacterial colonization in ticks.

**FIG 4:**
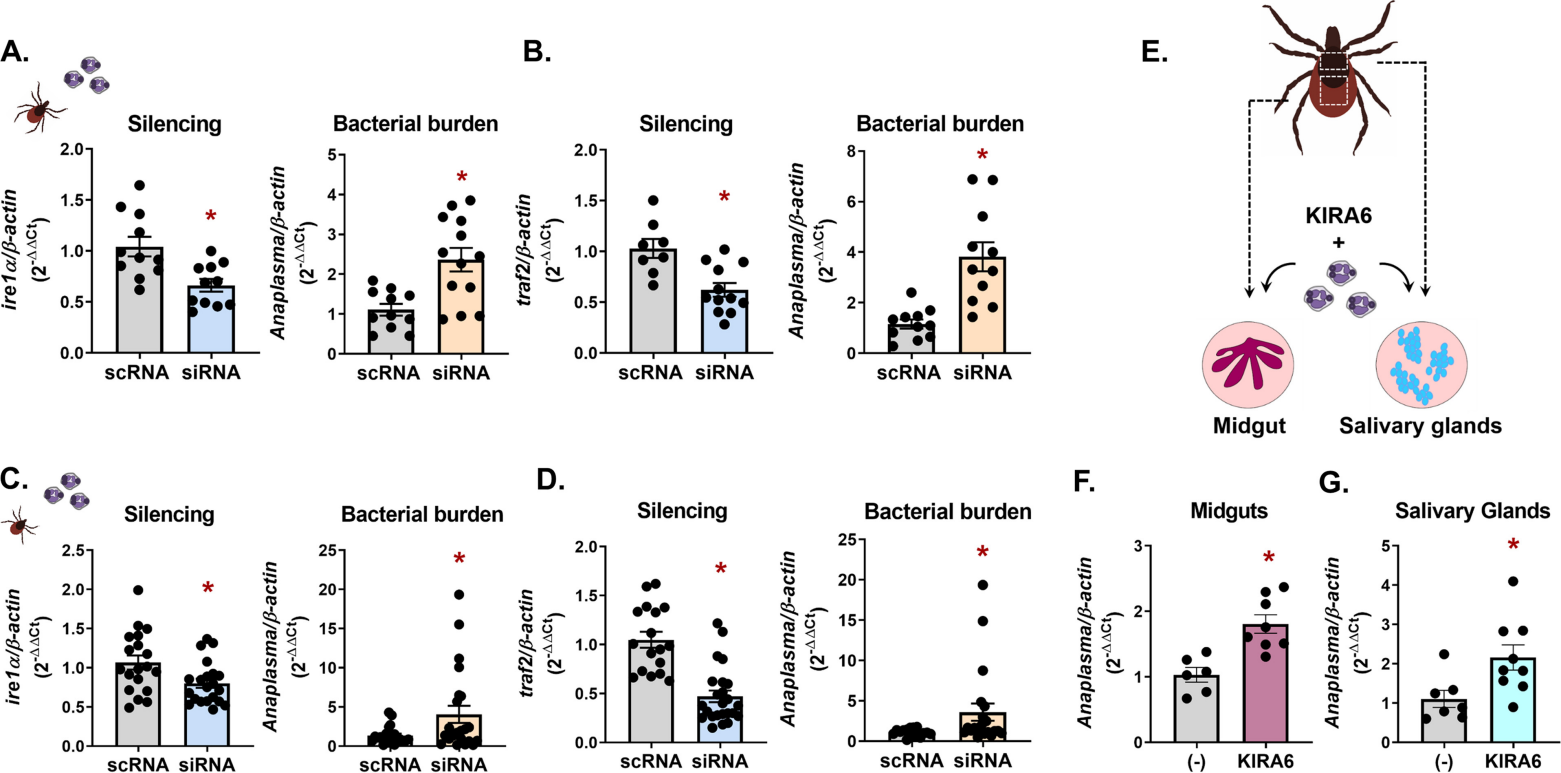
Vector competence for A. phagocytophilum is influenced by *Ixodes* IRE1α and TRAF2 at multiple life stages *in vivo.* I. scapularis (A and B) nymphs or (C and D) larvae had *ire1α* and *traf2* expression silenced through RNAi prior to feeding on A. phagocytophilum*-*infected mice. Silencing levels and bacterial load were measured in whole I. scapularis nymphs or larvae. (E) Schematic of *ex vivo* I. scapularis midgut and salivary gland cultures. (F and G) Midguts and salivary glands from I. scapularis adults were dissected, cultured, and treated with 1 μM KIRA6 (1 h) followed by A. phagocytophilum infection for 24 h. Silencing levels and A. phagocytophilum load (16S rRNA gene) were measured by qRT-PCR. Each point represents 1 tick, midgut, or pair of salivary glands (two technical replicates each); error bars show SEM. *, *P* < 0.05 (Welch’s *t* test). scRNA, scrambled RNA; siRNA, small interfering RNA.

We next determined whether the pathogen-restricting activity of *Ixodes* IRE1α and TRAF2 observed *in vitro* had similar impacts *in vivo*. To knock down gene expression, unfed I. scapularis nymphs were microinjected with small interfering RNA (siRNA) targeting *ire1α* and *traf2* or with a scrambled control RNA (scRNA). Nymphs were rested overnight and then fed to repletion on A. phagocytophilum-infected mice. Gene silencing and bacterial burden were both quantified by quantitative reverse transcriptase PCR (qRT-PCR). Similar to *in vitro* experiments, reducing the expression of *ire1α* and *traf2* led to an increase in A. phagocytophilum burdens in I. scapularis nymphs (Fig. 4A and B).

I. scapularis take a blood meal once per life stage, with ticks initially becoming infected during the larval phase (75). Since gene expression can vary depending on arthropod life stage (76–78), we examined the impact of IRE1α and TRAF2 on pathogen colonization in larvae. We silenced *ire1α* and *traf2* in I. scapularis larvae using a modified immersion protocol where ticks were submerged in siRNA or scrambled controls overnight (79). Following immersion, larvae were rested for 24 h before feeding to repletion on A. phagocytophilum-infected mice. Significant knockdown of *ire1α* and *traf2* was observed in siRNA-treated larvae with this method, which caused an increase in A. phagocytophilum numbers (Fig. 4C and D).

Soon after A. phagocytophilum is acquired, the bacteria migrate to the salivary glands, where they persist throughout the tick life cycle (75, 80, 81). To understand how IRE1α influences bacterial colonization in tick tissue subsets, we employed an *ex vivo* tick organ culture system (82, 83). Midguts and salivary glands from adult I. scapularis ticks were dissected and treated with the IRE1α inhibitor KIRA6 prior to infection with A. phagocytophilum (Fig. 4E). Similar to *in vitro* and *in vivo* findings, inhibiting the activity of IRE1α led to significantly higher A. phagocytophilum burdens in *ex vivo* salivary gland and midgut cultures (Fig. 4F and G), demonstrating that this signaling axis functionally restricts bacterial colonization in disparate tick tissues.

**FIG 5:**
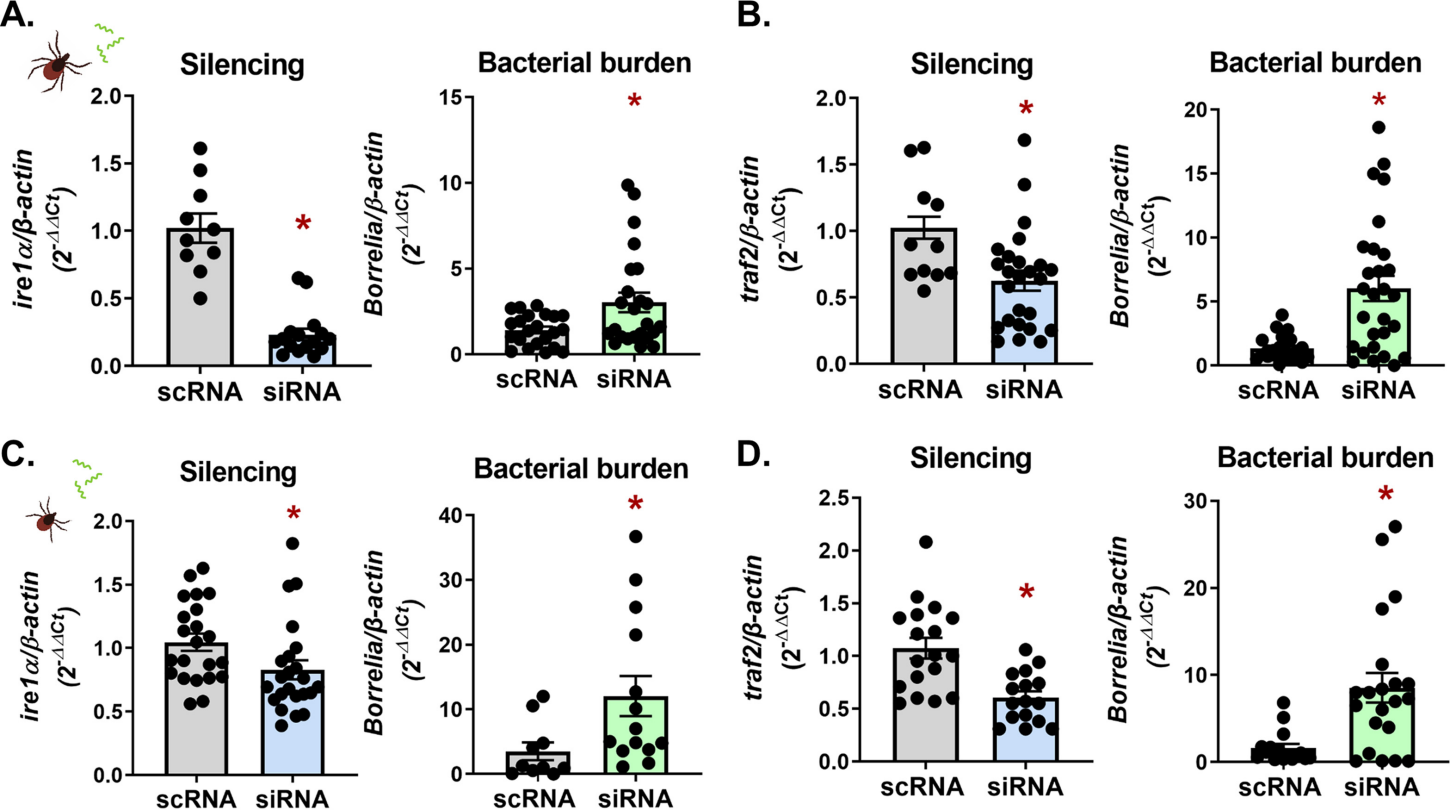
*Ixodes* IRE1α and TRAF2 restrict B. burgdorferi colonization *in vivo* at multiple tick life stages. RNAi silencing of *ire1α* and *traf2* in I. scapularis (A and B) nymphs or (C and D) larvae was performed prior to feeding on B. burgdorferi-infected mice. Silencing levels and B. burgdorferi (*flaB*) were measured in whole I. scapularis nymphs or larvae. Each point represents 1 tick (two technical replicates each); error bars show SEM. *, *P* < 0.05 (Welch’s *t* test). scRNA, scrambled RNA; siRNA, small interfering RNA.

We next asked whether the activity of IRE1α-TRAF2 signaling was restrictive to different tick-borne microbes, such as the Lyme disease-causing spirochete B. burgdorferi. Expression of *ire1α* and *traf2* was knocked down through RNAi in both I. scapularis nymphs and larvae using the methods described above, and ticks were fed to repletion on B. burgdorferi-infected mice. In agreement with the phenotype observed with A. phagocytophilum, significantly higher B. burgdorferi levels were observed in siRNA-treated ticks at both the nymph (Fig. 5A and B) and larval (Fig. 5C and D) stages. These data show that IRE1α-TRAF2 signaling is broadly responsive to multiple I. scapularis-transmitted pathogens and is functionally restrictive to microbial colonization during different tick life stages.

### The IMD pathway is triggered by IRE1α.

TRAF2 is a component of the mammalian TNFR network, which is functionally analogous to the arthropod IMD pathway. This parallel led us to ask whether the antimicrobial activity of the *Ixodes* IRE1α-TRAF2 axis operates through arthropod immunity. AMPs specific to the IMD pathway have not yet been identified in ticks. Instead, the *Drosophila* S2* cell line can be used as a surrogate model to quantify pathway-specific AMPs (32). To examine whether ER stress induces an immune response in the absence of microbes, we treated S2* cells with the UPR inducer thapsigargin. AMPs corresponding to the IMD pathway (diptericin, attacin A, and cecropin A2) (84) were significantly induced in a dose-dependent manner compared to unstimulated controls (Fig. S4A). In contrast, the Toll pathway AMP *IM1* (84–86) was not significantly different, demonstrating that ER stress leads to IMD pathway activation independent of microbial agonists.

**FIG 6:**
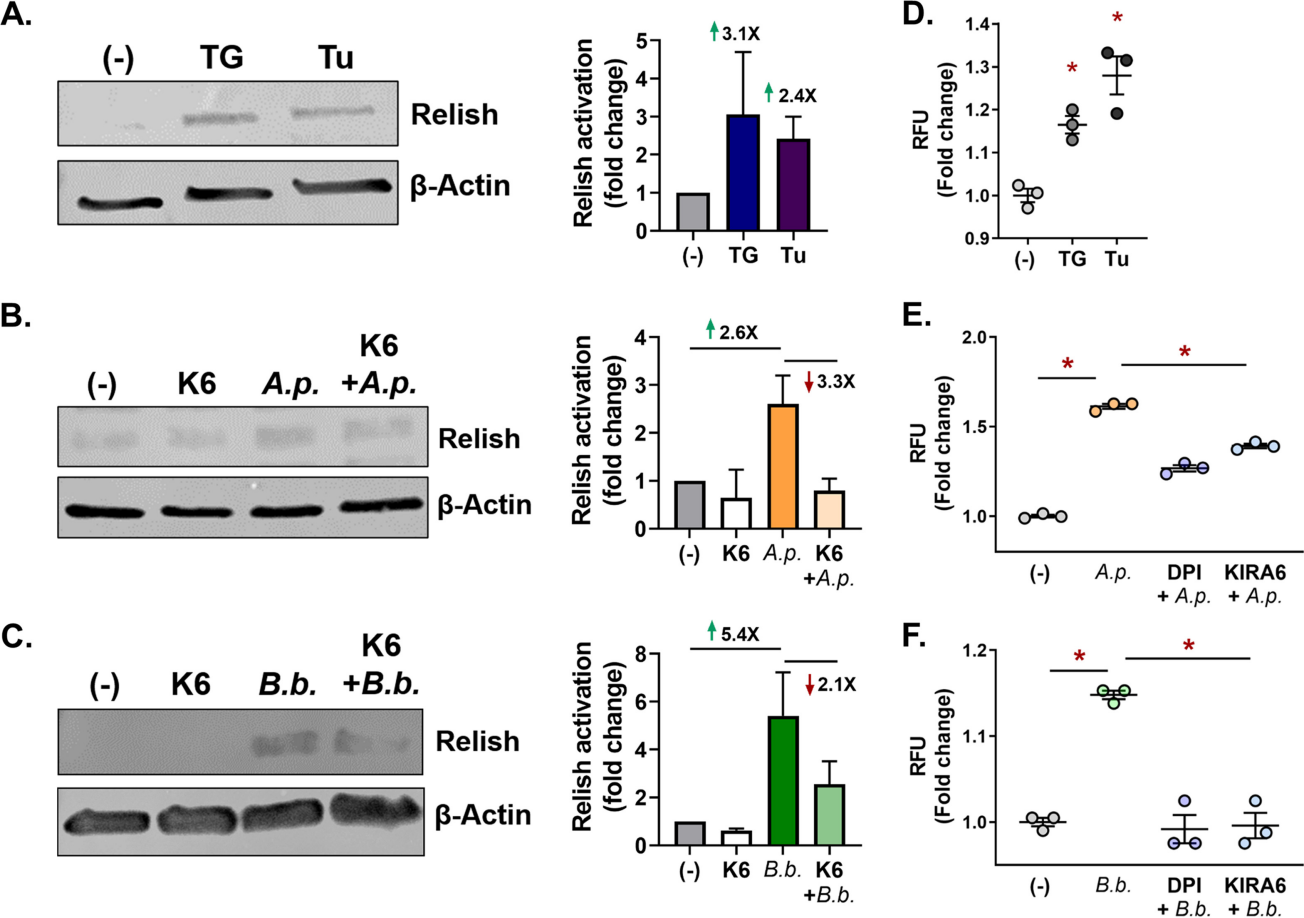
Infection-induced IMD pathway activation and ROS production function through IRE1α. (A to C) Relish immunoblot of ISE6 cells (A) stimulated for 1 h with thapsigargin (TG) or tunicamycin (Tu) or (B and C) pretreated with KIRA6 (K6) before (B) A. phagocytophilum (*A.p.*; MOI, 50) or (C) B. burgdorferi (*B.b.*; MOI, 50) infection (24 h). Immunoblots shown are representative of two or three biological replicates. Protein expression differences were quantified by ImageJ and are expressed as a ratio of Relish (~41 kDa) to the internal loading control, β-actin (45 kDa). (D to F) ROS assay with ISE6 cells (1.68 × 10^5^) stimulated with (D) thapsigargin (TG; 10 nM) or tunicamycin (Tu; 50 nM) or (E and F) ROS output from ISE6 cells pretreated with either DPI (5 μM) or KIRA6 (1 μM) for 1 h prior to (E) A. phagocytophilum or (F) B. burgdorferi infection. ROS was measured as RFU after 72 h. Data are representative of 3 biological replicates and 2 technical replicates; error bars show SEM. *, *P* < 0.05 (Student's *t* test). (-), vehicle control; DPI, diphenyleneidonium.

10.1128/mbio.00703-22.4 FIG S4 (Related to Fig. 6.) The UPR stimulates IMD pathway-associated antimicrobial peptides. (A) Indicated concentrations of thapsigargin (TG) were used to treat S2* cells (1 × 10^6^) for 6 h prior to examining gene expression differences. (B and C) S2* cells (1 × 10^6^) were pretreated with KIRA6 (1 h) before (B) A. phagocytophilum (*A.p.*; MOI, 50) or (C) B. burgdorferi (*B.b.*; MOI, 50) infection (6 h). Gene expression is relative to *rp49.* The dotted line denotes unstimulated controls. Data are representative of 4 or 5 biological replicates and two technical replicates. Download FIG S4, TIF file, 0.8 MB. Copyright © 2022 Sidak-Loftis et al. 2022 Sidak-Loftis et al. https://creativecommons.org/licenses/by/4.0/ This content is distributed under the terms of the Creative Commons Attribution 4.0 International license.

It is known that the IMD pathway is responsive to the tick-transmitted pathogens A. phagocytophilum and B. burgdorferi (32, 33). Since tick-borne microbes also activate the UPR (Fig. 1B and 2A) and ER stress induces the IMD network (Fig. S4A), we asked whether blocking IRE1α during infection would inhibit the IMD pathway. S2* cells that were treated with the IRE1α inhibitor KIRA6 prior to A. phagocytophilum or B. burgdorferi infection showed significantly reduced IMD pathway AMPs (Fig. S4B and C).

We next examined whether the tick IMD pathway underwent a similar UPR-driven activation event. Relish is the transcription factor associated with IMD pathway activation. Similar to what was observed in *Drosophila* S2* cells, ISE6 cells that were treated with UPR stimulator thapsigargin or tunicamycin showed an increase in Relish activation (Fig. 6A). We next asked if inhibiting IRE1α would block activation of the IMD pathway in ticks. ISE6 cells were stimulated with A. phagocytophilum and B. burgdorferi alone or were pretreated with the IRE1α inhibitor KIRA6 before infection. Pretreatment with KIRA6 resulted in a decline in Relish activation (Fig. 6B and C), indicating that infection-induced IMD pathway activation occurs through IRE1α. Collectively, our results provide strong evidence that the IRE1α-TRAF2 axis functions as an IMD pathway-activating mechanism.

### *Ixodes* IRE1α-TRAF2 signaling potentiates reactive oxygen species.

An immune mechanism complementary to the IMD pathway is the production of reactive oxygen species (ROS), which cause bactericidal damage to nucleic acids, proteins, and membrane lipids (16, 87, 88). Because B. burgdorferi and A. phagocytophilum are both sensitive to killing by ROS (89–92) and the mammalian UPR can lead to ROS production (53, 93), we investigated whether ROS can be induced by the *Ixodes* IRE1α-TRAF2 pathway. ISE6 cells were stimulated with either thapsigargin, tunicamycin, or a vehicle control and monitored for ROS with the fluorescent indicator 2′,7′-dichlorofluorescein diacetate. Pharmacological inducers caused significantly higher fluorescence, indicating that the tick UPR potentiates ROS (Fig. 6D). Infection with A. phagocytophilum and B. burgdorferi also elicited ROS production in tick cells (Fig. 6E and F). Pretreating ISE6 cells with the ROS-inhibiting agent diphenyleneidonium chloride (DPI) prior to infection reduced fluorescence, as expected. Importantly, blocking IRE1α activity with KIRA6 either reduced or completely mitigated ROS (Fig. 6E and F), demonstrating that infection-induced ROS production is potentiated by IRE1α.

### IRE1α-TRAF2 signaling restricts pathogens across tick vectors.

**FIG 7:**
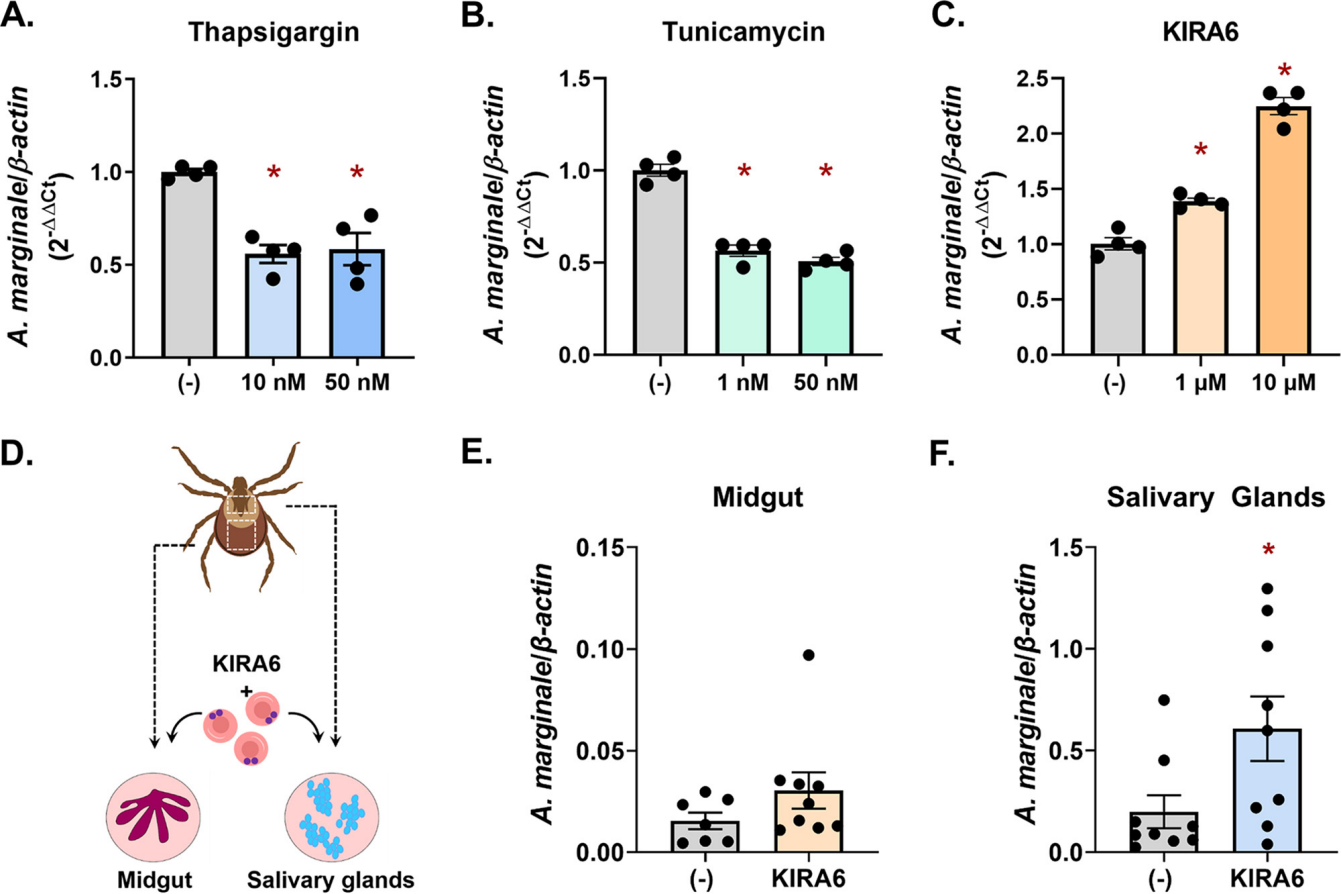
IRE1α and TRAF2-mediated pathogen restriction is conserved across arthropod vectors. DAE100 cells (5 × 10^5^) were treated with indicated concentrations of (A) thapsigargin, (B) tunicamycin, or (C) KIRA6 followed by infection with A. marginale (MOI, 50) for 18 h. *, *P* < 0.05 (Student's *t* test). (D) Schematic of *ex vivo D. andersoni* midgut and salivary gland cultures. (E and F) Midguts and salivary glands from *D. andersoni* adults were dissected, cultured, and treated with 1 μM KIRA6 (1 h) followed by A. marginale infection for 22 h. A. marginale (*rpoH*) was quantified by qRT-PCR and graphed relative to *β-actin.* *, *P* < 0.05 (Welch’s *t* test). Each point is representative of 1 tick, midgut, or pair of salivary glands (two technical replicates); error bars show SEM.

Since the UPR is conserved across eukaryotes, we explored the possibility that the microbe-restricting activity of IRE1α-TRAF2 signaling could functionally impact other arthropod vectors. *D. andersoni* ticks are important disease vectors that transmit several pathogens, including the obligately intracellular rickettsia A. marginale (94). When inducing the UPR in the *D. andersoni* tick cell line DAE100 with tunicamycin and thapsigargin (Fig. 7A and B) or blocking IRE1α with KIRA6 (Fig. 7C), we observed significant changes in A. marginale invasion and replication, comparable to what was observed with I. scapularis and A. phagocytophilum (Fig. 1C and D and 2C). Moreover, higher bacterial loads were also observed in *D. andersoni ex vivo* midgut and salivary gland cultures when IRE1α activity was blocked with KIRA6 (Fig. 7D to F). Altogether, this demonstrates that the microbe-restricting activity of IRE1α-TRAF2 signaling is conserved across tick species and is active against disparate pathogens, including intracellular bacteria (A. phagocytophilum and A. marginale) and extracellular spirochetes (B. burgdorferi).

## DISCUSSION

How arthropod immunity responds to infection is a fundamental factor influencing the ability of vectors to harbor and transmit pathogens (2–8). The IMD pathway is increasingly recognized as being divergent across species, with classically defined upstream regulators missing in many arthropod genomes (16–29, 31, 34). This suggests that an alternative activation mechanism exists. In this article, we demonstrate that the I. scapularis IMD pathway is initiated through the IRE1α-TRAF2 axis of the UPR. Colonization and replication of A. phagocytophilum and B. burgdorferi are restricted in ticks by *Ixodes* IRE1α and TRAF2 both *in vitro* and *in vivo*. Moreover, we show that IMD pathway activation and ROS production in response to A. phagocytophilum and B. burgdorferi are dependent on IRE1α activity and that this mode of antibacterial restriction is conserved across arthropods. Collectively, our findings provide an explanation for how the core IMD pathway is activated in the absence of canonical upstream regulators.

To our knowledge, this is the first time that cellular stress responses have been implicated in influencing vector competency. Why host cell stress responses are triggered by A. phagocytophilum and B. burgdorferi remains unclear. Ticks do not appear to suffer pathological consequences from the microbes they transmit. The connection between host cell stress and immune outcomes supports a model where transmissible pathogens would benefit most by decreasing infection-induced stress. This model is reenforced by the absence of common inflammatory PAMPs in many tick-transmitted pathogens. For example, all *Ixodes*-transmitted bacteria lack lipopolysaccharide (LPS) and DAP-PGN (95–98). B. burgdorferi flagella are housed in the periplasm, effectively shielded from recognition by host cells (99). During coevolution with ticks, *Ixodes*-transmitted pathogens may have lost inflammatory PAMPs with the benefit of reducing cellular stress and host responses, thereby promoting persistence and transmission. Nevertheless, our data show that A. phagocytophilum and B. burgdorferi exert at least some stress on ticks. Since immune responses are energetically costly to the host (100, 102), we speculate that the tick response is tuned to match the level of threat imposed by infection, ultimately striking a balance that conserves resources and preserves tick fitness (9, 101).

Our findings indicate a mechanism of IMD pathway activation that deviates from the classically defined paradigm where pattern recognition receptors (PRRs) sense bacterially derived PAMPs. Both intracellular and extracellular pathogens exert stress on the host, which can be caused by secreted toxic by-products, competition for nutrients, and/or physical damage to host cells/organ systems (50). For example, B. burgdorferi is an extracellular spirochete and an extreme auxotroph that lacks many central metabolic pathways (102, 103). To get around this limitation, it parasitizes purines (104), amino acids (105), cholesterol (106, 107), long-chain fatty acids (108, 109), carbon sources (110), and other metabolites (111) from the host. A. phagocytophilum is obligately intracellular and parasitizes amino acids and cholesterol from the host, in addition to manipulating host cell processes with secreted effectors (112–117). From this perspective, both microbes cause stress to the host by competing for a finite amount of resources and disturbing normal cellular processes. Indeed, our evidence shows that tick-transmitted microbes stimulate the UPR and are restricted by its activity. Although cellular stress responses detect and respond to stress, they are not necessarily specific to types of stressors and instead respond by monitoring macromolecular threats to the cell (40, 41, 118). This more generalized signal widens the infection-sensing scope of possibility and reduces the requirement for an array of specific immune receptors. In this regard, a wide variety of stimuli would converge on a common immune outcome. Since the UPR is an evolutionarily conserved mechanism across eukaryotes (36–38), it is feasible that UPR-initiated immunity is a fundamental mode of pathogen sensing and host defense against a broad array of infectious organisms.

The absence of upstream IMD pathway regulators appears to be a shared trait among chelicerates and hemimetabolous insects (16–25, 27–33, 101, 120). Considering this observation, classically defined IMD pathway-initiating molecules may have evolved with holometabolous insects (those with complete metamorphosis), such as *Drosophila* and mosquitos, which undergo dramatic tissue remodeling between life stages (119, 121). The classically described IMD pathway is speculated to have arisen as a defense mechanism against microbiome-resident bacteria that are liberated with midgut breakdown during metamorphosis (31). In contrast, arthropods with incomplete metamorphosis, such as ticks and hemimetabolous insects, undergo some tissue remodeling during molting, but immature stages generally resemble adults in terms of morphology (119, 122, 123). Since these arthropods are phylogenetically more ancient than holometabolous insects (119, 121, 124–126), it is possible that UPR activation of core IMD signaling molecules is ancestral to the classically defined pathway in *Drosophila*. Furthermore, our data support the idea that this network is conserved across arthropods. Specifically, IRE1α-mediated IMD pathway activation was observed both in *Drosophila* cells (Fig. S4B and C) and in two species of ticks (Fig. 6 and 7). Collectively, this suggests that the UPR-IMD signaling network may have evolved in a common ancestor of hexapods and chelicerates.

**FIG 8:**
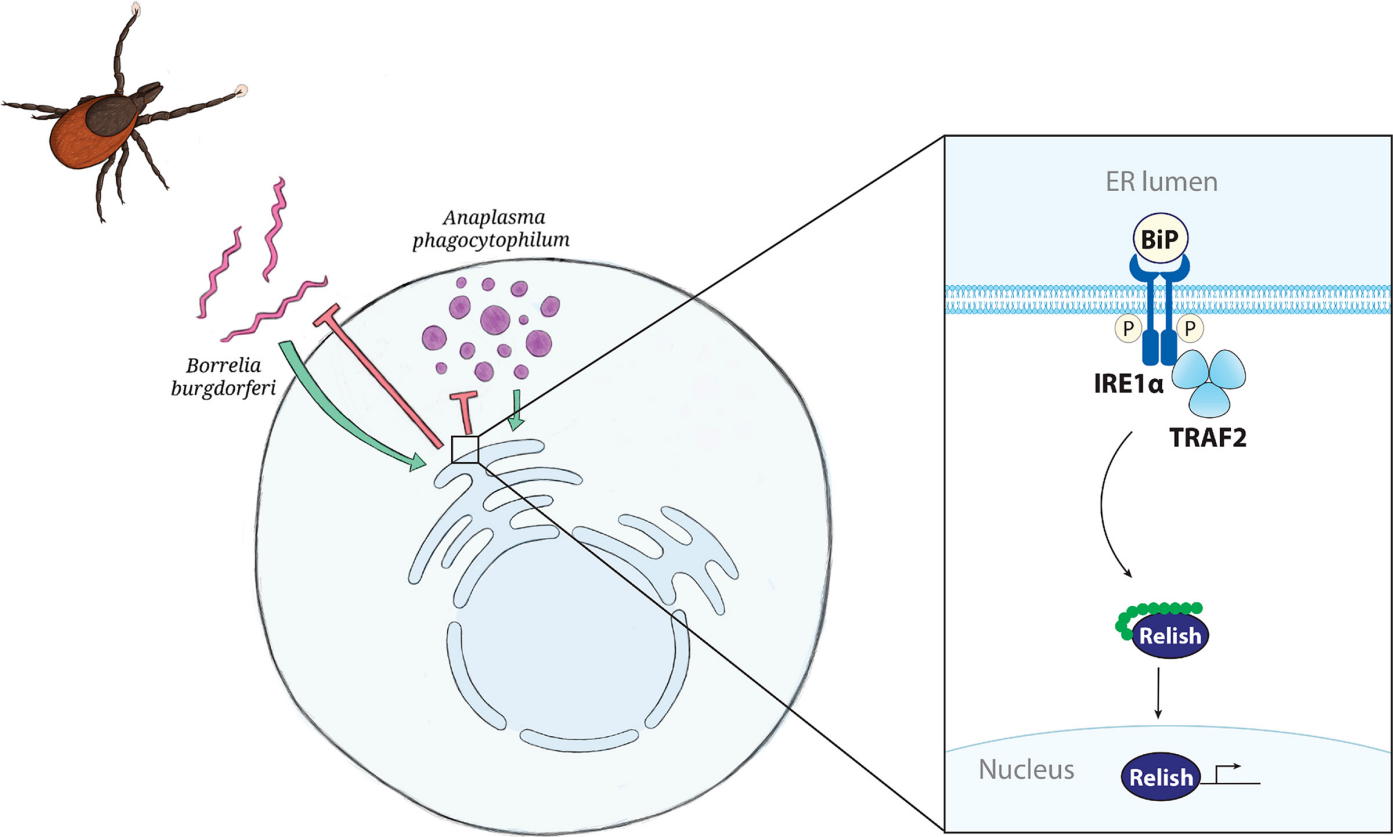
The UPR triggers the IMD pathway in ticks. The tick-borne bacteria A. phagocytophilum and B. burgdorferi stimulate the UPR in I. scapularis ticks. IRE1α is activated by phosphorylation (P) and pairs with TRAF2. This signaling axis induces the IMD pathway, Relish activation, and antimicrobial responses that restrict pathogen colonization.

In summary, we have discovered a linkage between cellular stress responses and arthropod immunity where the IRE1α-TRAF2 signaling axis initiates the IMD pathway (Fig. 8). The previous “orphaned” status of the IMD pathway in ticks was a perception that arose from comparative studies with the insect model organism *Drosophila*. This revelation underscores the importance of studying fundamental processes outside model organisms, which may be valuable for determining concepts that could be basally applicable across species. Our findings are conceptually important given that the IMD pathway widely impacts vector competence in many arthropods. With this commonality, one can envision a scenario where a conserved network across species may be an attractive target for future transmission intervention strategies.

## MATERIALS AND METHODS

### Bacteria and animal models.

Escherichia coli cultures were grown in lysogeny broth (LB) supplemented with ampicillin at 100 μg μL^−1^. Cultures were grown overnight at 37°C with shaking between 230 and 250 rpm.

A. phagocytophilum strain HZ was cultured in HL60 cells with Roswell Park Memorial Institute (RPMI) 1640 medium supplemented with 10% heat-inactivated fetal bovine serum (FBS; Atlanta Biologicals; S11550) and 1× Glutamax (Gibco; 35050061). Cells were maintained between 1* × *10^5^ and 1* × *10^6^ mL^−1^ at 37°C in 5% CO_2_. A. phagocytophilum was enumerated as previously described (32). Briefly, the percentage of infected cells is multiplied by the average number of microcolonies (termed “morulae”) per cell (5), the average bacteria per morula (19), and the average amount of bacteria typically recovered from the isolation procedure (50%). Host cell-free A. phagocytophilum was isolated by syringe lysis with a 27-gauge needle as previously described (3).

B. burgdorferi B31 (strain MSK5 [127]) was grown in modified Barbour-Stoenner-Kelly II (BSK-II) medium supplemented with 6% normal rabbit serum (NRS; Pel-Freez; 31126-5) at 37°C in 5% CO_2_ (70, 127). Spirochete density and growth phase were monitored by dark-field microscopy. Prior to infection, plasmid profiles of all B. burgdorferi cultures were screened by PCR, as described previously (127).

Uninfected I. scapularis ticks were provided by the Biodefense and Emerging Infectious Diseases (BEI) Research Resources Repository from the National Institute of Allergy and Infectious Diseases (NIAID) (www.beiresources.org) at the National Institutes of Health (NIH) or from Oklahoma State University (Stillwater, OK, USA). Ticks were maintained in a 23°C incubator with 16/8-h light/dark photoperiods and 95 to 100% relative humidity. C3H/HeJ mice were purchased from The Jackson Laboratory, and C57BL/6 mice were obtained from colonies maintained at Washington State University. Six- to ten-week-old male mice were used for all experiments. C57BL/6 mice were infected intraperitoneally with 1 × 10^7^ host cell-free A. phagocytophilum organisms. C3H/HeJ mice were inoculated intradermally with 1 × 10^5^ low-passage-number B. burgdorferi. All mice were confirmed for infection status prior to tick placement by collecting 25 to 50 μL of blood from the lateral saphenous vein of each mouse 7 days postinfection. A. phagocytophilum burdens were enumerated by quantitative PCR (*16s* relative to mouse *β-actin* [128, 129]). B. burgdorferi-infected blood was subcultured in BSK-II medium and examined for the presence of spirochetes by dark-field microscopy (130, 131). Experiments involving mice were carried out according to guidelines and protocols approved by the American Association for Accreditation of Laboratory Animal Care (AAALAC) and by the Office of Campus Veterinarian at Washington State University (Animal Welfare Assurance A3485-01). The animals were housed and maintained in an AAALAC-accredited facility at Washington State University in Pullman, WA. All procedures were approved by the Washington State University Biosafety and Animal Care and Use Committees.

### D. melanogaster and tick cell cultures.

D. melanogaster S2* cells were cultured with Schneider’s *Drosophila* medium (Gibco; 21720024) supplemented with 10% heat-inactivated FBS (Sigma; SH30070) and 1× Glutamax. Cells were maintained in T75 culture flasks (Corning; 353136) at 28°C.

The I. scapularis tick cell line ISE6 was cultured at 32°C and 1% CO_2_ in L15C-300 medium supplemented with 10% heat-inactivated FBS (Sigma; F0926), 10% tryptose phosphate broth (TPB; BD; B260300) and 0.1% lipoprotein bovine cholesterol (LPBC; MP Biomedicals; 219147680) (35). The *D. andersoni* tick cell line, DAE100, was maintained at 34°C and cultured in L15B medium supplemented with 5% FBS, 10% TPB, and 1% LPBC as previously described (132, 133).

### Polyacrylamide gel electrophoresis and Western blotting.

Protein concentrations were quantified using bicinchoninic acid (BCA) assays per manufacture protocol (Pierce; 23225). Fifty micrograms of protein per sample was separated on a 4 to 15% MP TGX precast cassette (Bio-Rad; 4561083) at 100 V for 1 h 25 min before being transferred to a polyvinylidene difluoride (PVDF) membrane. Membranes were blocked with 5% milk in PBS-T (1× phosphate-buffered saline containing 0.1% Tween 20) for 1 to 2 h at room temperature before being incubated at 4°C overnight with a primary antibody in PBS-T with 5% bovine serum albumin (BSA) or 0.5% to 5% milk. Primary antibodies used for immunoblotting are as follows: anti-phospho-IRE1α (Abcam; ab124945; 1:1,000), anti-Relish (gift from Joao Pedra; 1:500), antiactin (Sigma; A2103; 1:1,000), anti-HA (Pierce; 26183, 1:1,000), and horseradish peroxidase (HRP)-conjugated anti-FLAG (Sigma; A8592; 1:500). Secondary antibodies were applied for 1 to 2 h at room temperature and are as follows: goat anti-rabbit–HRP (Abcam; ab97051; 1:5,000), donkey anti-rabbit–HRP (Thermo Fisher Scientific; A16023; 1:2,000), rabbit anti-mouse–HRP (Bio-Rad; STAR13B; 1:2,000), and RecG protein–HRP (Thermo Fisher Scientific; 101223; 1:2,000). Blots were visualized with enhanced chemiluminescence (ECL) Western blotting substrate (Thermo Fisher Scientific; 32106). If necessary, blots were stripped with Western blot stripping buffer (Thermo Fisher Scientific; 21059) for 15 to 20 min at room temperature with shaking. Protein expression differences were quantified by ImageJ as described by Hossein Davarinejad (http://www.yorku.ca/yisheng/Internal/Protocols/ImageJ.pdf) and are expressed as a ratio of the target protein to the internal loading control.

### Plasmid construction.

Both *Ixodes* IRE1α and TRAF2 were codon optimized for expression in human cell lines (GenScript). Primers (Table S1) were used to amplify full-length I. scapularis *ire1α* for cloning into pCMV/hygro-Negative Control Vector (SinoBiological; CV005) with HindIII sites. Full-length I. scapularis *traf2* was amplified and cloned into pCMV-HA (New MCS) vector (received as a gift from Christopher A. Walsh; Addgene plasmid number 32530) using XhoI and EcoRV. All constructs were confirmed by sequencing (Eurofins Genomics).

10.1128/mbio.00703-22.1 TABLE S1 Primer sequences. Download TABLE S1, PDF file, 0.1 MB. Copyright © 2022 Sidak-Loftis et al. 2022 Sidak-Loftis et al. https://creativecommons.org/licenses/by/4.0/ This content is distributed under the terms of the Creative Commons Attribution 4.0 International license.

### Maintenance and transfection of HEK 293T cells.

HEK 293T cells were cultured in Dulbecco’s modified Eagle medium (DMEM; Sigma; D6429) supplemented with 10% heat-inactivated FBS (Atlanta Biologicals; S11550) and 1× Glutamax. Cells were maintained in T75 culture flasks (Corning; 353136) at 37°C in 5% CO_2_. For transfection, 1 × 10^6^ HEK 293T cells were seeded into 6-well plates and allowed to attach overnight. The following day, cells were transfected with 2.5 μg of pCMV-TRAF2-HA and/or pCMV-IRE1α-FLAG plasmid DNA using 10 μL of Lipofectamine 2000 (Invitrogen; 11668027) in Opti-MEM I reduced-serum medium (Gibco; 31985062). After 5 h, medium containing the plasmid-Lipofectamine 2000 complex was removed and replaced with complete DMEM for 48 h at 33°C and 5% CO_2_. The transfected cells were lysed with 500 μL of 25 mM Tris-HCl (pH 7.4), 150 mM NaCl, 1% NP-40, 1 mM EDTA, 5% glycerol with 1× protease and phosphatase inhibitor cocktail (Thermo Scientific; 78440) for 15 min on ice.

### Coimmunoprecipitation assay.

*Ixodes* IRE1α-FLAG and TRAF2-HA expression was validated by immunoblotting whole-cell lysates with anti-FLAG–HRP (Sigma; A8592; 1:500) and anti-HA (Pierce; 26183; 1:1,000). After protein expression was confirmed, cross-linked agarose beads (anti-FLAG M2 [Sigma; A2220] and anti-HA [Pierce; 26181]) were washed twice with TBS (50 mM Tris, 150 mM NaCl; pH 7.5) and incubated with lysis buffer at 4°C for 1 h. Approximately 1 to 2 mg of cell lysate was combined with 80 μL (packed volume) of cross-linked agarose beads and incubated overnight at 4°C. Beads were washed 3 times with TBS, and protein was eluted by boiling in 50 μL of 4× Laemmli buffer for 5 min. Protein interactions were evaluated by immunoblotting as described above.

### Template-based homology modeling of *Ixodes* TRAF2 and the RNase/kinase domain of IRE1α.

A BLAST search in the Protein Data Bank (PDB) using the *Ixodes* TRAF2 sequence returned the candidate template crystal structure of the TRAF-C domain from human TRAF2 (39.64% sequence identity). The human TRAF2 crystal structure (PDB code 1CA9) was used as a reference for building the homology model of the TRAF-C domain and part of the coiled-coil domain for *Ixodes* TRAF2 (residues 176 to 357) in SWISS-MODEL (71, 134). QMEANDisCo was used to obtain a quality score, which defines how well the homology model aligns to reference structures in the PDB. Scores closer to 1 indicate that the homology model matches other reference structures well (135). Quality assessment of the TRAF2 homology model in QMEANDisCo gave a score of 0.69. The GalaxyRefine server was used to then further refine the *Ixodes* TRAF2 homology model, which increased the quality score in QMEANDisCo to 0.71 (136).

A PDB BLAST search for *Ixodes* IRE1α returned the candidate template crystal structure of the RNase/kinase domain from human IRE1α (62.20% sequence identity). A homology model for the cytosolic RNase/kinase domain of tick IRE1α (residues 525 to 944) was built using the crystal structure of the RNase/kinase domain from humans (PDB code 6URC) with SWISS-MODEL (72, 134). Quality assessment of the tick IRE1α homology model in QMEANDisCo gave a score of 0.78.

### Prediction-driven docking of *Ixodes* TRAF2 and the RNase/kinase domain of IRE1α.

A consensus interface predictor, CPORT (consensus prediction of interface residues in transient complexes), was used to assign residues at the interface of *Ixodes* TRAF2 and the IRE1α RNase/kinase domain (69). Predicted residues were used to define the docking interface between *Ixodes* TRAF2 and IRE1α for docking in HADDOCK 2.2 (74). The docked model was immersed in a solvent shell using the TIP3P water model, and a short 300-K molecular dynamics (MD) simulation was run to optimize side chains and improve interaction energetics (74). The cluster with the lowest Z score was chosen for further analysis. Docking models were then screened based on salt bridge interactions at the docking interface, and the model with the best chemical complementarity was used in the final analysis. PyMOL version 2.2.3 was used for all distance measurements of salt-bridge interactions (<4-Å cutoff) (PyMOL molecular graphics system; Schrodinger, LLC).

### ROS assay.

ISE6 cells were seeded at a density of 1.68* × *10^5^ cells per well in a black-walled, clear-bottom 96-well plate (Thermo Scientific; 165305) with L15C-300 medium. The cells were maintained under the growth conditions described above for the length of the experiments. All wells were treated for 1 h with 10 μM 2′,7′-dichlorofluorescein diacetate (DCF-DA; Sigma; D6883) in Ringer buffer (155 mM NaCl, 5 mM KCl, 1 mM MgCl_2_ · 6H_2_O, 2 mM NaH_2_PO_4_ · H_2_O, 10 mM HEPES, and 10 mM glucose) (53) alone or with 5 μM diphenyleneidonium chloride (DPI; Sigma; D2926), 1 μM KIRA6 (Cayman Chemical; 19151), or 0.1% dimethyl sulfoxide (DMSO). Buffer was removed; cells were washed with room temperature 1× PBS and incubated for 72 h in L15C-300 alone or with A. phagocytophilum (multiplicity of infection [MOI], 200), B. burgdorferi (MOI, 200), 10 nM thapsigargin (TG; Sigma; T9033), or 50 nM tunicamycin (Tu; Sigma; T7765). Fluorescence was measured at 504 nm (excitation) and 529 nm (emission). Data are graphed as fold change of relative fluorescence units (RFU) normalized to the negative control, with standard errors of the means (SEM).

### Pharmacological treatments, RNAi silencing, and qRT-PCR.

ISE6 cells were seeded at 1 × 10^6^ cells per well and DAE100 cells were seeded at 5 × 10^5^ cells per well in a 24-well plate and pretreated with KIRA6, thapsigargin, or tunicamycin for indicated times and concentrations prior to infection. Cells were infected with A. phagocytophilum (ISE6) or A. marginale (DAE100) at an MOI of 50 for 18 h before collection in TRIzol (Invitrogen; 15596026). For DAE100 experiments, all incubations occurred at 34°C in a BD Campy Bag with no GasPak. RNA was extracted using the Direct-zol RNA microprep kit (Zymo; R2062). cDNA was synthesized from 300 to 500 ng total RNA with the Verso cDNA synthesis kit (Thermo Fisher Scientific; AB1453B). Bacterial burden and gene silencing were assessed by qRT-PCR with iTaq universal SYBR green Supermix (Bio-Rad; 1725125). Cycle conditions were as recommended by the manufacturer.

For transfection experiments, siRNAs and scrambled controls (scRNAs) were synthesized following directions from the Silencer siRNA construction kit (Invitrogen; AM1620). siRNA or scRNA (3 μg) was used to transfect 1 × 10^6^ ISE6 cells overnight with 2.5 μL of Lipofectamine 2000. Cells were infected with A. phagocytophilum (MOI, 50) for 18 h before being collected in TRIzol. RNA was isolated and transcripts were quantified by qRT-PCR as described above. All data are expressed as means and SEM.

### *Ex vivo* I. scapularis and *D. andersoni* organ culture.

Ten male and female unfed adult I. scapularis ticks were surface sterilized with continuous agitation in 10% benzalkonium chloride (Sigma; 12060) for 10 min, washed twice with sterile water, dried on sterile filter paper under aseptic conditions, and transferred to a sterile tube. Midgut and salivary glands were excised on a microscope slide in a pool of sterile 1× PBS with 100 IU mL^−1^ penicillin and 100 μg mL^−1^ streptomycin (Gibco; 15140122). Tissues were placed in individual wells of a 96-well plate (Costar; 3595) with 100 μL of L15C-300 and incubated at 32°C with 1% CO_2_. Tissues were treated with 1 μM KIRA6 or 1% DMSO for 1 h before the addition of 1 × 10^6^ A. phagocytophilum. At 24 h postinfection, samples were collected following the addition of 100 μL of TRIzol. Tissues were homogenized using QIAshredder columns (Qiagen; 79654) according to the manufacturer’s instructions prior to RNA extraction and qRT-PCR analysis, performed as described above.

Twenty male unfed adult *D. andersoni* ticks were surface sterilized and dissected as described above. Tissues were placed in individual wells of a 96-well plate with 100 μL of L15B. Tissues were pretreated with KIRA6 or vehicle control (DMSO) as stated above prior to the addition of 1* × *10^6^ A. marginale for 22 h. Samples were collected and processed as described above with qRT-PCR standard curves. All data are expressed as means and SEM.

### RNAi silencing in nymphs and larvae.

I. scapularis nymphs were microinjected as described previously (32, 35). Ten-microliter Drummond microdispensers (DrummondSci; 3000203G/X) were drawn to fine-point needles using a Narishige PC-100 micropipette puller. I. scapularis nymphs were microinjected with 25 nl of siRNA or scRNA (~1,000 ng/μL) into the anal pore using a Drummond Nanoject III nanoliter injector (DrummondSci; 3000207). Ticks were allowed to rest overnight before being placed between the ears and on the back of an infected mouse. Each group was placed on a single mouse and fed to repletion (5 to 7 days). Nymphs were flash frozen with liquid nitrogen, individually crushed with a plastic pestle, and suspended in TRIzol for RNA extraction.

I. scapularis larvae were prechilled at 4°C for 5 min. Approximately 150 larvae were transferred to a 1.5-mL tube with 40 to 50 μL of either siRNA or scRNA (~1,000 ng/μL). The tubes were centrifuged at 3,000 × *g* for 5 min to encourage submersion of the larvae in the dsRNA and were then incubated overnight at 15°C. The following day, ticks were dried and rested overnight before being placed on mice to feed until repletion (3 to 7 days). Larvae were flash frozen in liquid nitrogen and individually crushed with a plastic pestle. TRIzol was added before proceeding to RNA isolation and qRT-PCR analysis, performed as stated above.

### *xbp1* PCR and agarose gel electrophoresis.

RNA was isolated from both ISE6 cells or replete I. scapularis nymphs (uninfected, A. phagocytophilum infected, or B. burgdorferi infected). ISE6 cells were treated with either 0.5 μM thapsigargin or A. phagocytophilum at an MOI of 50. Cells were collected 1, 3, 8, and 24 h posttreatment in TRIzol. RNA was isolated and cDNA synthesized as previously described. The cleavage status of *xbp1* was assessed via PCR using DreamTaq Green PCR Mastermix (Thermo Scientific; K1082) and the *xbp1* primers (Table S1) with the cycling protocol recommended by the manufacturer. Samples were analyzed using a 3% agarose (Thermo Fisher; BP160) gel in 1× Tris-borate-EDTA (TBE; Thermo Fisher; BP1333) with 0.5 μg mL^−1^ of ethidium bromide (Thermo Fisher; BP102) and imaged with a ProteinSimple AlphaImager HP system.

### UPR and IMD gene expression profiling.

Untreated I. scapularis nymphs were fed to repletion on A. phagocytophilum-infected mice or uninfected mice and frozen. The expression levels of UPR genes were assessed in individual ticks by qRT-PCR as previously described. Primers specific for *bip*, *ire1α*, *xbp1*, and *traf2* were used (not shown). Data are expressed as means and SEM.

D. melanogaster S2* cells (1 × 10^6^) were seeded in Schneider’s medium with 1 μM 20-hydroxyecdysone to prime the IMD pathway, as previous reported (137). Cells were treated with indicated concentrations of thapsigargin for 6 h or with 10 μM KIRA6 for 1 h prior to infection with A. phagocytophilum (MOI, 50) or B. burgdorferi (MOI, 50) for 6 h. Samples were collected in TRIzol, and RNA was isolated. IMD pathway- and Toll pathway-specific AMPs were quantified by qRT-PCR with primers as described above.

### Gene alignment.

UPR gene sequences were identified by querying the I. scapularis genome with Homo sapiens protein sequences using NCBI (National Center for Biotechnology Information) protein BLAST. Human sequences include BiP (NP_005338.1), IRE1α (NP_001424.3), TRAF2 (NP_066961.2), and XBP1 (NP_005071.2). Human and tick sequences were aligned using Jalview (138). Shaded regions indicate amino acid physiochemical property conservation (Fig. S1A to D). EMBL-EBI (European Bioinformatics Institute) Pfam 34.0 was used to identify and annotate protein domains (139).

### Statistical analysis.

*In vitro* experiments were performed with 3 to 5 replicates. *In vivo* experiments involved the use of 10 to 20 ticks. Data were expressed as means and SEM and analyzed with either unpaired Student's *t* test or Welch’s *t* test. Calculations and graphs were created with GraphPad Prism version 9.0. A *P* value of <0.05 was considered statistically significant.
