# Correction for Sidak-Loftis et al., “The Unfolded-Protein Response Triggers the Arthropod Immune Deficiency Pathway”

**DOI:** 10.1128/mbio.02922-22

**Published:** 2022-11-03

**Authors:** Lindsay C. Sidak-Loftis, Kristin L. Rosche, Natasha Pence, Jessica K. Ujczo, Joanna Hurtado, Elis A. Fisk, Alan G. Goodman, Susan M. Noh, John W. Peters, Dana K. Shaw

## AUTHOR CORRECTION

Volume 13, no. 4, e00703-22, 2022, https://doi.org/10.1128/mbio.00703-22. Page 15, Acknowledgments, line 6: “to D.K.S.” should read “to D.K.S. and 1R01GM138592-01 to J.W.P.”

