# Supplementary figures and images for: The Unfolded-Protein Response Triggers the Arthropod Immune Deficiency Pathway
Source: mBio. 2022 Jul 18;13(4):e00703-22. doi: 10.1128/mbio.00703-22 (PMC9426425; doi:10.1128/mbio.00703-22)

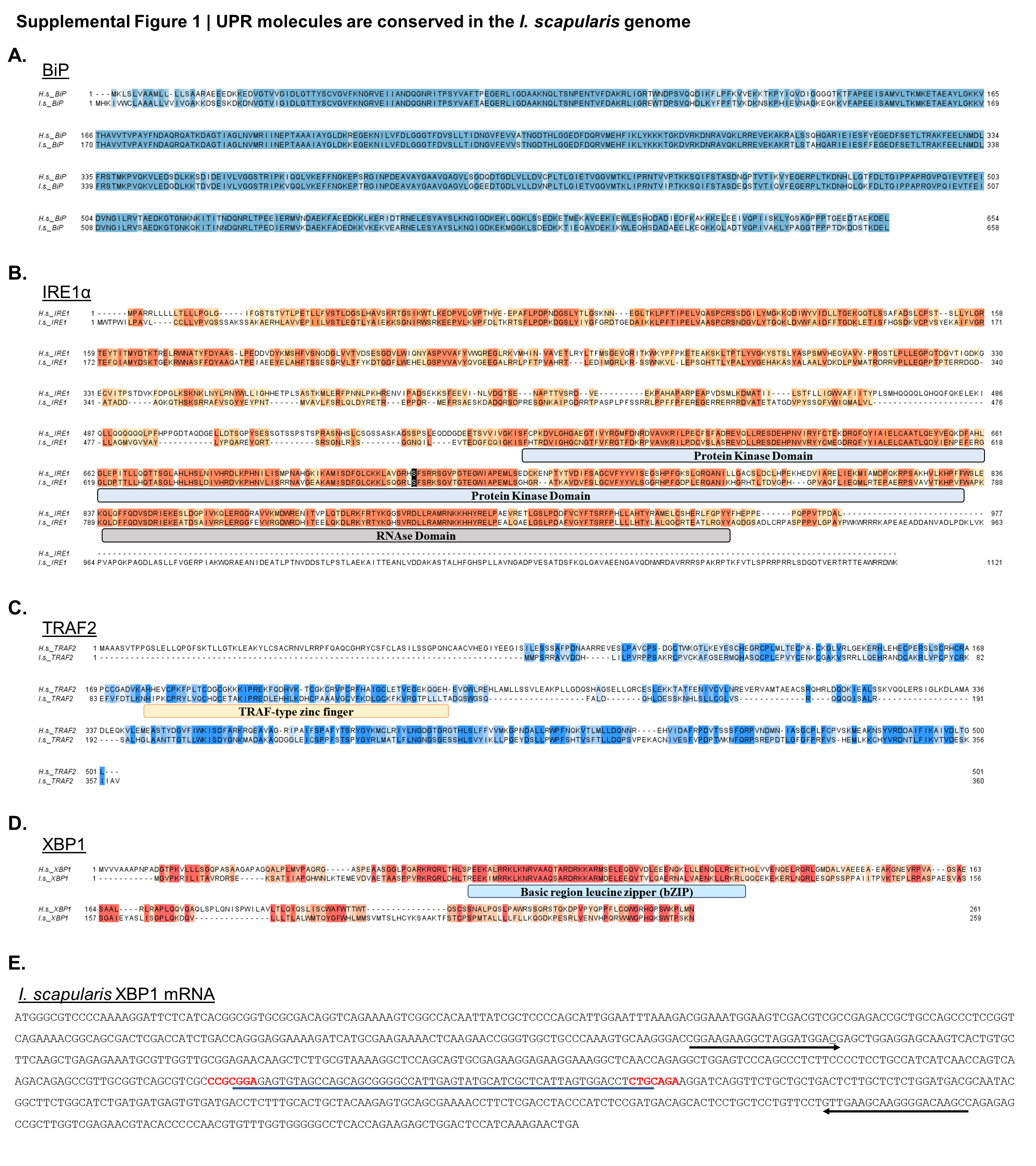

Supplement: FIG S1 [file mbio.00703-22-s0001.tif]

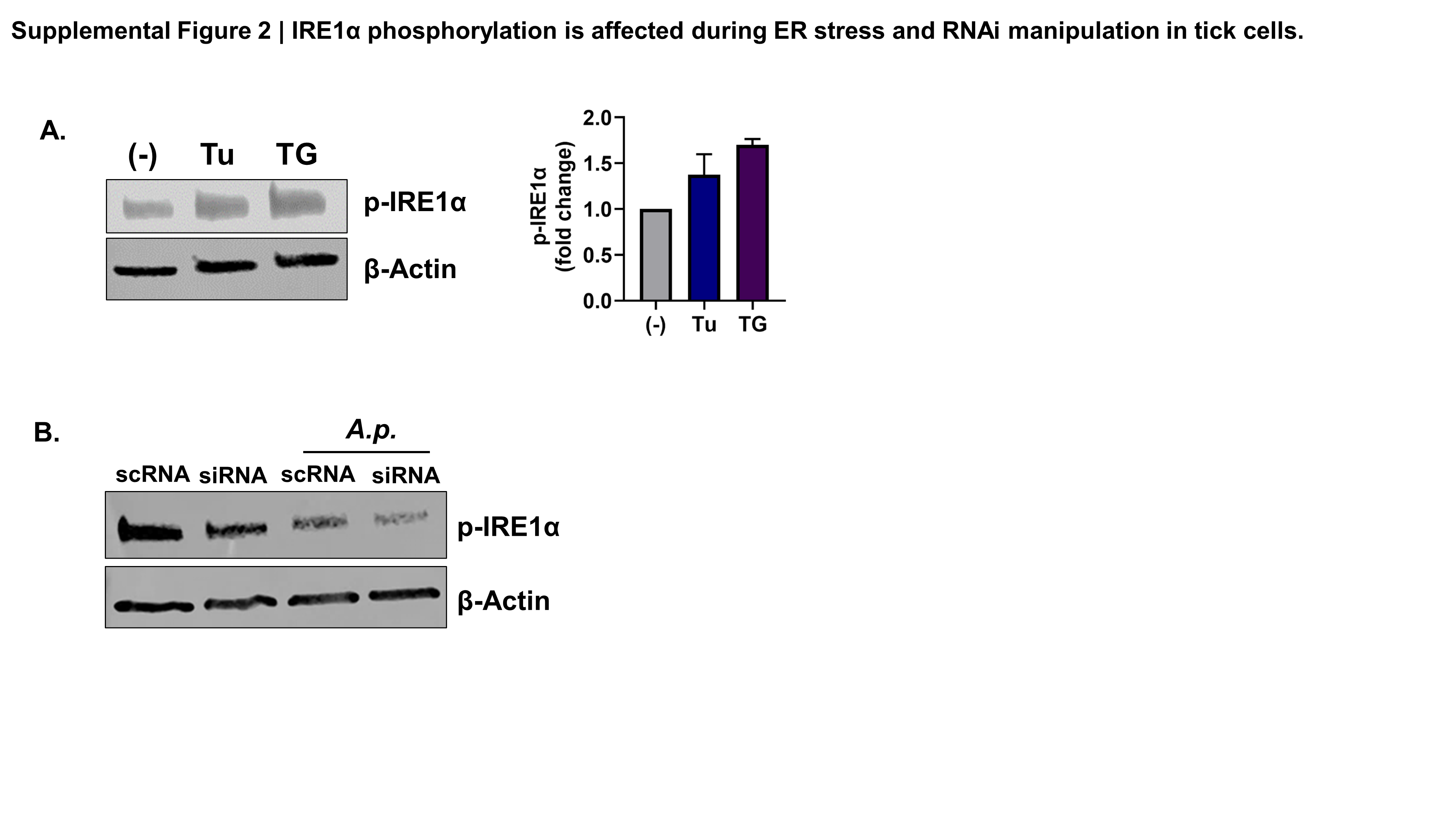

Supplement: FIG S2 [file mbio.00703-22-s0002.tif]

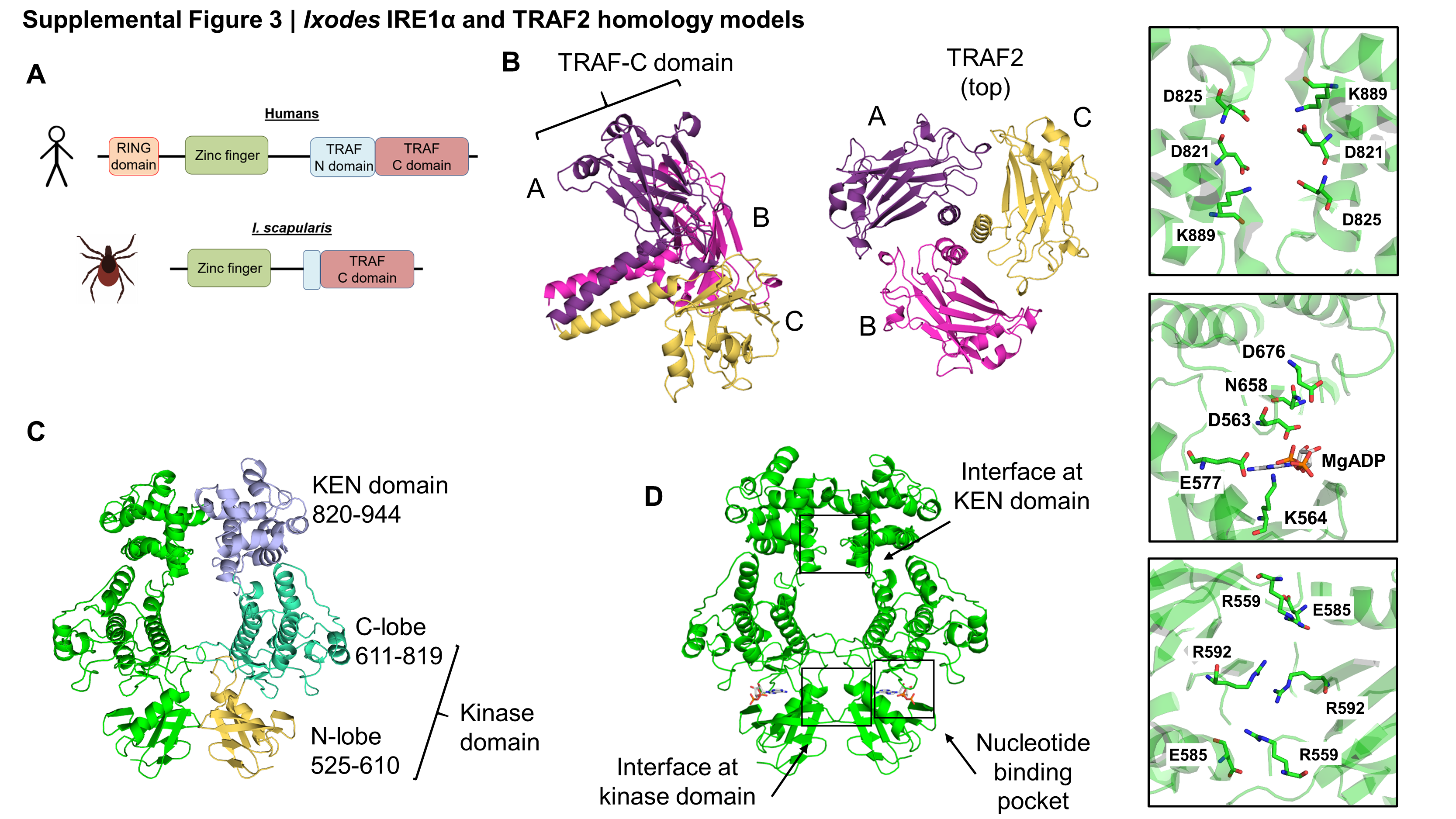

Supplement: FIG S3 [file mbio.00703-22-s0003.tif]

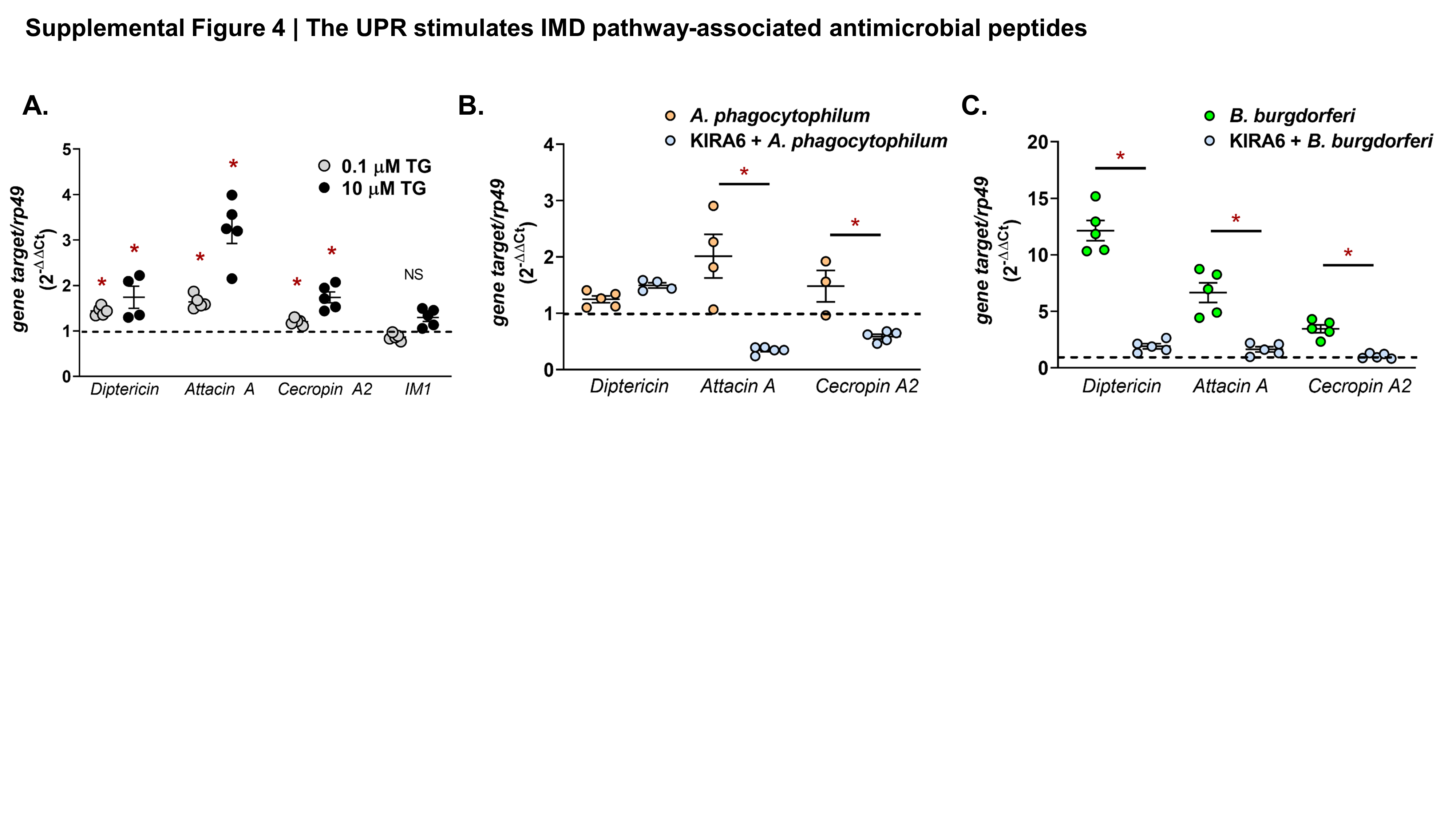

Supplement: FIG S4 [file mbio.00703-22-s0004.tif]
