# Supplementary material for: The Unfolded-Protein Response Triggers the Arthropod Immune Deficiency Pathway
Source: mBio. 2022 Jul 18;13(4):e00703-22. doi: 10.1128/mbio.00703-22 (PMC9426425; doi:10.1128/mbio.00703-22)
Supplement: TABLE S1 [file mbio.00703-22-s0005.pdf]

Supplemental Table 1

Name	Target gene	Primer Sequences
Mus musculus β-Actin (qRT-PCR)	XM_030254057.1	F 5'-ACGCAGAGGGAAATCGTGCGTGAC-3'<br>R 5'-ACGCGGGAGGAAGAGGATGCGGCAGTG-3'
Anaplasma phagocytophilum 16S (qRT-PCR)	NC_007797	F 5'-CCCTAAGGCCTTCCTCACTC-3'<br>R 5'-CAGCCACACTGGAACTGAGA-3'
Anaplasma marginale rpoH (qRT-PCR)	AAV86624.1	F 5'-GCGGATGCCCATAGGTTGGT-3'<br>R 5'-TACGTCGCAAGCCTGAAGCC-3'
Borrelia burgdorferi FlaB (qRT-PCR)	MN954474.1	F 5'-TTGCTGATCAAGCTCAATATAACCA-3'<br>R 5'-TTGAGACCCTGAAAGTGATGC-3'
Dermacenter andersoni β-Actin (qRT-PCR)	EF488512.2	F 5'-CGCCTCCTCCTCTTCTCTG-3'<br>R 5'-TGTAGGTGGTCTCGTGATG-3'
Ixodes scapularis Actin (qRT-PCR)	XM_029977298.1	F 5'-GCCGGGACCTTACAGACTATC-3'<br>R 5'-CACGGACAATTTCACGCTCG-3'
Ixodes scapularis IRE1α (qRT-PCR)	XM_029972190.1	F 5'-GAGAAGGCCATCTTCGTCGG-3'<br>R 5'-GAGTAGCCTGGGCAGCATAG-3'
Ixodes scapularis TRAF2 (qRT-PCR)	XM_029977983.1	F 5'-CCGCGAAAAGAACAGCTTAC-3'<br>R 5'-TACCACGTTGGACTCCTTCC-3'
Ixodes scapularis BiP (qRT-PCR)	XM_002433611.2	F 5'-ATCGTGTGGTGTCTAGCGG-3'<br>R 5'-CGATACCGATGACTGTGCCG-3'
Ixodes scapularis Xbp1 (PCR)	XM_002410360.1	F 5'-CGGAAGAAGGCTAGGATGGAC-3'<br>R 5'-GCTTGTCCCCTTGCTTCAAC-3'
Ixodes scapularis Xbp1 (qRT-PCR)	XM_002410360.1	F 5'- AGAGAAATGCGTTGGTTGCG-3'<br>R 5'- GATGATGGCAGGAGGGGAAG-3'
Drosophila melanogaster Diptericin (qRT-PCR)	NM_057460.4	F 5'-CCGCAGTACCCACTCAATCT-3'<br>R 5'-ACTGCAAAGCCAAAACCATC-3'
Drosophila melanogaster Attacin (qRT-PCR)	NM_079021.5	F 5'-CACAATGTGGTGGGTCAGG-3'<br>R 5'-GGCACCATGACCAGCATT-3'
Drosophila melanogaster Cecropin (qRT-PCR)	NM_079850.4	F 5'-GGACAATCGGAAGCTGGTT-3'<br>R 5'-TGTGCTGACCAACACGTTC-3'
Drosophila melanogaster RP49 (qRT-PCR)	NM_001144655.3	F 5'-AAGCTAGCCCAACCTGGTTG-3'<br>R 5'-GTGCGCTTCTTCACGATCT-3'
Drosophila melanogaster IM1 (qRT-PCR)	NM_137475.3	F 5'-TCCACTGTCGCCCGATCC-3'<br>R 5'-CTTGGGTTGAAACTTCCTACTTGC-3'
Ixodes scapularis IRE1α _siRNA_382	XM_029972190.1	F 5'-AACCTGAAATAGCGGAGGAATCCTGTCTC-3'<br>R 5'-AAATTCCTCCGCTATTTCAGGCCTGTCTC-3'
Ixodes scapularis IRE1α_ scRNA	N/A	F 5'-AAGACGGATAAGCCGAGTTAACCTGTCTC-3'<br>R 5'-AATTAACTCGGCTTATCCGTCCCTGTCTC-3'
Ixodes scapularis TRAF2_siRNA_1422	XM_029977983.1	F 5'-AAGCAGATCCAGAGGCAATATCCTGTCTC-3'<br>R 5'-AAATATTGCCTCTGGATCTGCCCTGTCTC-3'
Ixodes scapularis TRAF2_ scRNA	N/A	F 5'-AAGGAGTCAAGTCGCTACAAACCTGTCTC-3'<br>R 5'-AATTTGTAGCGACTTGACTCCCCTGTCTC-3'
Ixodes scapularis BiP_siRNA_184	XM_002433611.1	F 5'-AAGGAAACCGTATCACGCCATCCTGTCTC-3'<br>R 5'-AAATGGCGTGATACGGTTTCCCCTGTCTC-3'
Ixodes scapularis BiP_scRNA	N/A	F 5'-AAGCCGACCTAAGGACTCTAACCTGTCTC-3'<br>R 5'-AATTAGAGTCCTTAGGTCGGCCCTGTCTC-3'
pCMV-IRE1α-FLAG	XM_029972190.1	F 5'-AAAAGCTTATGTGGACCCCATGGATCCTG-3'<br>R 5'-AAAAGCTTTTTCCAGTCACGGCGCCAAGC-3'
pCMV-TRAF2-HA	XM_029977983.1	F 5'-AACTCGAGATGCCGAGCCGTCGCGCT-3'<br>R 5'-AAGATATCTTACACGGCAATGATTTTGCTCTCATCC-3'
